# Regulation of the double-stranded RNA response through ADAR1 licenses metaplastic reprogramming in gastric epithelium

**DOI:** 10.1172/jci.insight.153511

**Published:** 2022-02-08

**Authors:** José B. Sáenz, Nancy Vargas, Charles J. Cho, Jason C. Mills

**Affiliations:** 1 Division of Gastroenterology, Department of Medicine, Washington University in St. Louis School of Medicine, St. Louis, Missouri, USA.; 2 Section of Gastroenterology and Hepatology, Department of Medicine;; 3 Department of Pathology and Immunology; and Department of Molecular and Cellular Biology, Baylor College of Medicine, Houston, Texas, USA.

**Keywords:** Cell Biology, Gastroenterology, Cell stress, Innate immunity

## Abstract

Cells recognize both foreign and host-derived double-stranded RNA (dsRNA) via a signaling pathway that is usually studied in the context of viral infection. It has become increasingly clear that the sensing and handling of endogenous dsRNA is also critical for cellular differentiation and development. The adenosine RNA deaminase, ADAR1, has been implicated as a central regulator of the dsRNA response, but how regulation of the dsRNA response might mediate cell fate during injury and whether such signaling is cell intrinsic remain unclear. Here, we show that the ADAR1-mediated response to dsRNA was dramatically induced in 2 distinct injury models of gastric metaplasia. Mouse organoid and in vivo genetic models showed that ADAR1 coordinated a cell-intrinsic, epithelium-autonomous, and interferon signaling–independent dsRNA response. In addition, dsRNA accumulated within a differentiated epithelial population (chief cells) in mouse and human stomachs as these cells reprogrammed to a proliferative, reparative (metaplastic) state. Finally, chief cells required ADAR1 to reenter the cell cycle during metaplasia. Thus, cell-intrinsic ADAR1 signaling is critical for the induction of metaplasia. Because metaplasia increases cancer risk, these findings support roles for ADAR1 and the response to dsRNA in oncogenesis.

## Introduction

Pathogen- and damage-associated molecular patterns (PAMPs, DAMPs) trigger innate immune responses by engaging pattern recognition receptors (PRRs; ref. 1). An essential innate immune pathway in vertebrates is the response to double-stranded RNA (dsRNA), a known DAMP/PAMP (2). One arm of the dsRNA response involves the recognition of foreign (i.e., viral) or host dsRNA by the cytosolic PRRs DDX58 and MDA5 (interferon induced with helicase C domain 1; IFIH1), leading to downstream activation of type I interferons (IFNs; e.g., IFN-α, IFN-β) and IFN-stimulated genes (ISGs; refs. 3, 4). Secreted type I IFNs can also bind cell surface–associated type I IFN receptors (i.e., IFNAR1/2) and can further amplify the innate immune response in a paracrine and/or autocrine manner (5, 6). Although the host response to dsRNA has routinely been studied in the context of an antiviral response (7–9), it is becoming clear that host-derived dsRNA can also trigger innate immune signaling (10, 11).

Cells have evolved mechanisms to distinguish self from non-self dsRNA (12), and the failure to suppress reactivity to self dsRNA can lead to aberrant autoimmunity (13, 14). A key regulator in the intracellular response to dsRNA is the adenosine RNA deaminase ADAR1, which functions as a rheostat for this pathway via RNA editing–dependent and –independent mechanisms (15). More importantly, ADAR1 is required during embryonic development, as *Adar1^–/–^* mice die by E12.5 and exhibit a significant upregulation of ISGs and defects in liver development and erythropoiesis (15–17). These mice can be rescued to attain adulthood when *Ifih1* is also deleted (18). ADAR1 is also required for the maintenance of fetal and adult hematopoietic stem cells (19, 20). Taken together, these findings suggest that the ability to sense and handle dsRNA extends beyond simply generating an antiviral state but also has critical implications for cell fate and differentiation. A major unresolved issue is how the cell-intrinsic (i.e., independent of exogenous immune signals, DAMPs, and PAMPs) regulation of dsRNA determines cell fate during homeostasis or during injury.

In addition to being instrumental to embryogenesis (18) or to the differentiation of adult tissue stem cells (19), cell identity switches are a critical aspect of the cellular reprogramming that occurs when cells attempt to regenerate after severe tissue injury (21). Sustained injury in the stomach, for example, leads to a gradual loss of acid-secreting parietal cells from the gastric corpus (22) and a reorganization of corpus units into a pattern known as pyloric metaplasia (23) (herein referred to as metaplasia). Metaplasia is characterized in part by the reprogramming of postmitotic chief cells at the base of the gastric gland into a proliferating population of spasmolytic polypeptide-expressing metaplastic (SPEM) cells (24), which can be histologically identified in mice by the coexpression of mucous neck cell markers (e.g., *Griffonia simplicifolia*, or GSII) and chief cell markers (e.g., gastric intrinsic factor, or GIF) at the gland base (25). Postmitotic chief cells become SPEM cells via a stepwise cellular reorganization program known as paligenosis, which first involves a degradation of the secretory machinery, followed by a reexpression of metaplastic markers prior to cellular proliferation (26). A critical step in paligenosis is the progression of SPEM cells from a stage where they express metaplastic genes to the final stage, where they reenter the cell cycle to proliferate. Recent evidence suggests that appropriately regulating the epithelial metaplastic response, in particular the proliferation of metaplastic cells, has implications for cellular survival and for the development of gastric cancer (27, 28), the third leading cause of cancer-related deaths worldwide (29). A role for dsRNA signaling in metaplasia or epithelial reprogramming has not been previously reported to our knowledge; however, ADAR1 has been implicated as a potential oncogene across various gastrointestinal organs (30, 31), including the stomach (32, 33). How ADAR1 increases gastric cancer risk is unclear.

In this study, we establish a potentially novel and previously unexplored role for the ADAR1-mediated dsRNA response in epithelial injury. We demonstrate that the response to dsRNA was substantially upregulated across 2 established, distinct, in vivo models of epithelial injury and metaplasia in the stomach. Consistent with this, dsRNA accumulated within metaplastic gastric epithelium in mice and in humans. We also find that the activation of the dsRNA response was independent of IFNAR1 signaling in vivo, indicating that it is not a function of immune signaling but rather cell intrinsic. Finally, we highlight a cell-autonomous, immune cell–independent role for ADAR1 in regulating epithelial reprogramming during injury: loss of ADAR1 impaired the chief cells’ intrinsic ability to proliferate during paligenosis. Thus, dsRNA signaling, acting in part through ADAR1, plays a cell-intrinsic role in cellular reprogramming during metaplasia and may help explain how ADAR1 can act as an oncogene in certain situations.

## Results

### Double-stranded RNA accumulates during gastric metaplasia.

**Figure 1:**
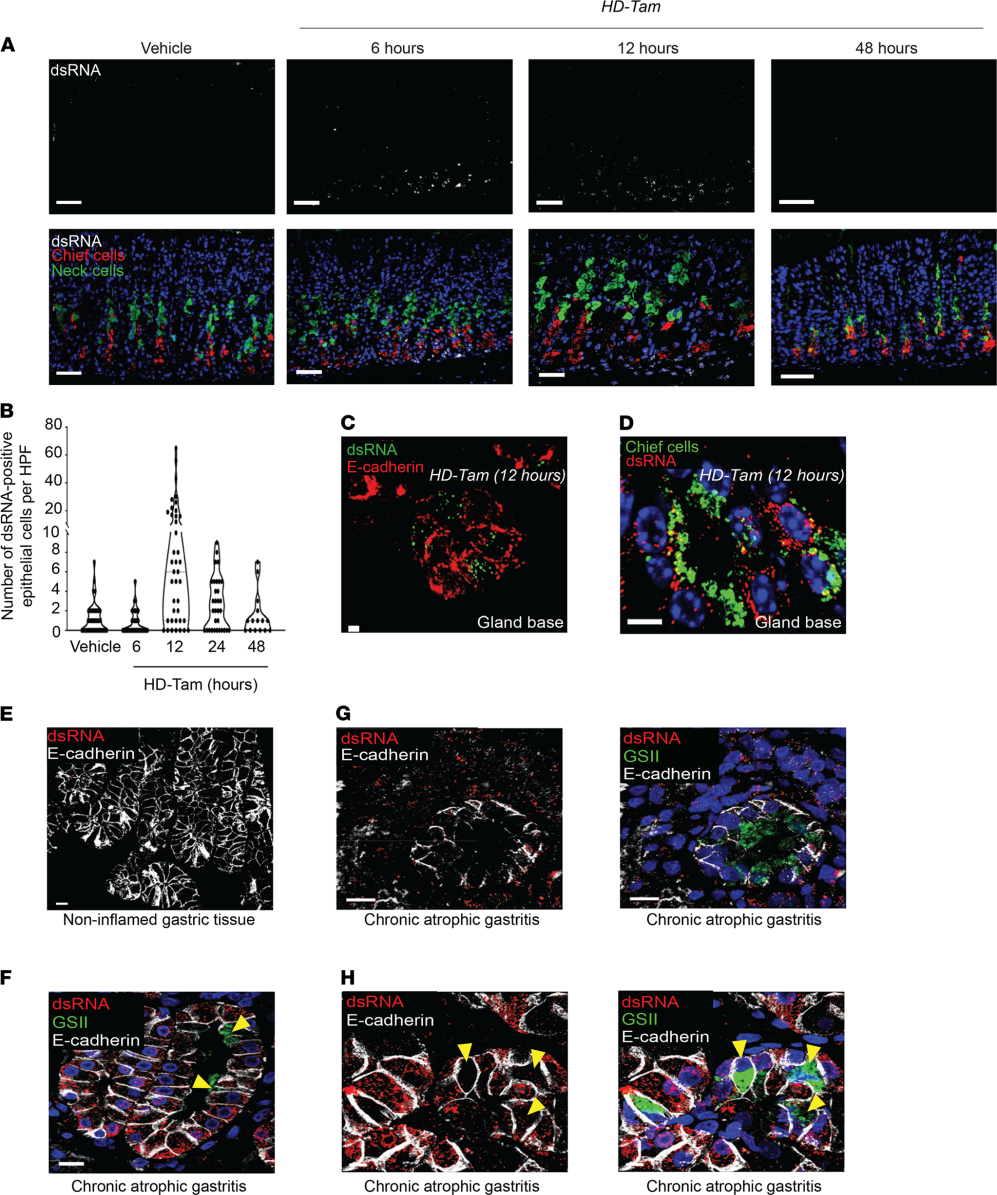
Double-stranded RNA accumulates within inflamed gastric epithelium. (**A**) Representative gastric corpus sections from wild-type mice treated with HD-Tam for the indicated times. Isolated dsRNA signal is shown in the top panels and merged images at the bottom. Scale bars, 20 μm. Images are representative of 3 mice per time point. (**B**) Quantification of epithelial cells harboring dsRNA. Each data point represents the total dsRNA/E-cadherin double-positive cells within a randomly selected HPF from 3 mice per time point. (**C** and **D**) Representative mouse corpus gland bases following 12 hours of HD-Tam demonstrating the accumulation of dsRNA within epithelial cells (**C**; red) and within chief cells (**D**; highlighted by GIF in green). Scale bars, 10 μm. (**E**–**H**) dsRNA expression in human gastric epithelium. (**F**) A gastric corpus biopsy from an *H*. *pylori*–positive patient with chronic atrophic gastritis demonstrates dsRNA (red) within inflamed epithelial cells (outlined in white) and largely absent from metaplastic cells (yellow arrowheads) showing GSII positivity (green). (**G**) A metaplastic corpus gland base from an *H*. *pylori*–positive patient with chronic atrophic gastritis demonstrates a relative paucity of dsRNA within metaplastic epithelium (marked in green; right panel). (**H**) This metaplastic corpus gland base from an *H*. *pylori*–positive patient with chronic atrophic gastritis shows hybrid features, with dsRNA accumulating in inflamed epithelium but largely excluded from metaplastic cells. Yellow arrowheads point to metaplastic cells at gland bases that express the mucous neck cell marker, GSII (green), and show a relative paucity of dsRNA. No dsRNA is seen in uninflamed gastric epithelial cells in an *H*. *pylori*–negative, uninflamed gastric biopsy (**E**). For **G** and **H**, isolated dsRNA and E-cadherin signals are shown in the left panels. Scale bars, 10 μm. HD-Tam, high-dose tamoxifen; GIF, gastric intrinsic factor.

To test whether dsRNA signaling is involved in cell fate decisions during injury, we relied on a previously validated model for acutely and synchronously inducing gastric metaplasia in mice (high-dose tamoxifen, HD-Tam; refs. 34, 35). We observed a time-dependent accumulation of dsRNA within gastric epithelium undergoing metaplastic changes (Figure 1A). While dsRNA was absent from gastric epithelium at homeostasis, its accumulation within epithelial cells (Figure 1C) and chief cells (Figure 1D) peaked within 12 hours of HD-Tam treatment and subsequently returned to baseline levels by 48 hours (Figure 1, A and B). The peak in epithelial dsRNA preceded the induction of metaplastic genes, as the dsRNA-positive cells did not show the coexpression of markers for chief and mucous neck cell genes that are pathognomonic for the transition to metaplasia (Figure 1A) (36). To confirm that the dsRNA observed within murine metaplastic epithelium was not due to uptake of viral RNA within the stomach, we intraperitoneally injected gnotobiotic (germ-free) mice with HD-Tam and found that the accumulation of dsRNA followed similar kinetics as specific pathogen–free mice (Supplemental Figure 1, A and B; supplemental material available online with this article; https://doi.org/10.1172/jci.insight.153511DS1).

We next examined a series of 150 biopsies from human gastric corpus and selected those demonstrating chronic gastritis as determined by the diagnostic pathologist (Table 1). As a control, we also examined a series of resection specimens from sleeve gastrectomies, all of which showed no histologic evidence of inflammation. We have previously shown that, in atrophic gastritis, there are regions that exhibit transitions between normal chief cell and SPEM type metaplastic cell morphology. Such transitions recapitulate the progression of metaplasia seen in mouse models (26, 37–39). As in control mouse chief cells, dsRNA was rarely found within normal chief cells in the sleeve gastrectomy specimens (Figure 1E). However, similar to murine chief cells early after HD-Tam injury, human chief cells that still exhibited a chief cell phenotype in human atrophic gastritis accumulated dsRNA, both within basal chief cells as well as within the surrounding inflammatory infiltrate (Figure 1, F and G). However, in more established metaplastic cells, dsRNA was less abundant than in surrounding inflammatory cells (Figure 1G). In basal units with hybrid morphology, dsRNA abundance showed an inverse correlation with the degree of labeling with the lectin GSII, a marker of SPEM metaplasia: those cells with abundant GSII (i.e., metaplastic cells) had the least amount of dsRNA (Figure 1, F and H, and Table 1).

### The dsRNA response is conserved across multiple models of gastric metaplasia.

As the pattern of dsRNA accumulation in murine chief cells following HD-Tam injury appeared to faithfully model metaplastic transitions in humans, we hypothesized that the response to dsRNA was part of the conserved reprogramming process that converts normal chief cells into proliferative metaplastic cells (26, 28). To further test this hypothesis, we used a mouse model for inducing gastric metaplasia in a way that more closely mimics human metaplasia. Whereas our HD-Tam model described above induces rapid, synchronous, and reversible metaplasia with mild inflammatory cell infiltrate (34), chronic infection with the stomach-adapted bacterium *H. pylori*, the biggest risk factor for the development of human gastric adenocarcinoma (40), also induces gastric metaplasia in mice. Just as in humans, *H. pylori* infection in mice induces metaplasia in a more indolent, multifocal, and asynchronous manner than HD-Tam, often with a robust inflammatory component (35, 41). As expected, both injury models induced metaplastic epithelial changes, despite the divergent relative role of the immune response (Supplemental Figure 2).

**Figure 2:**
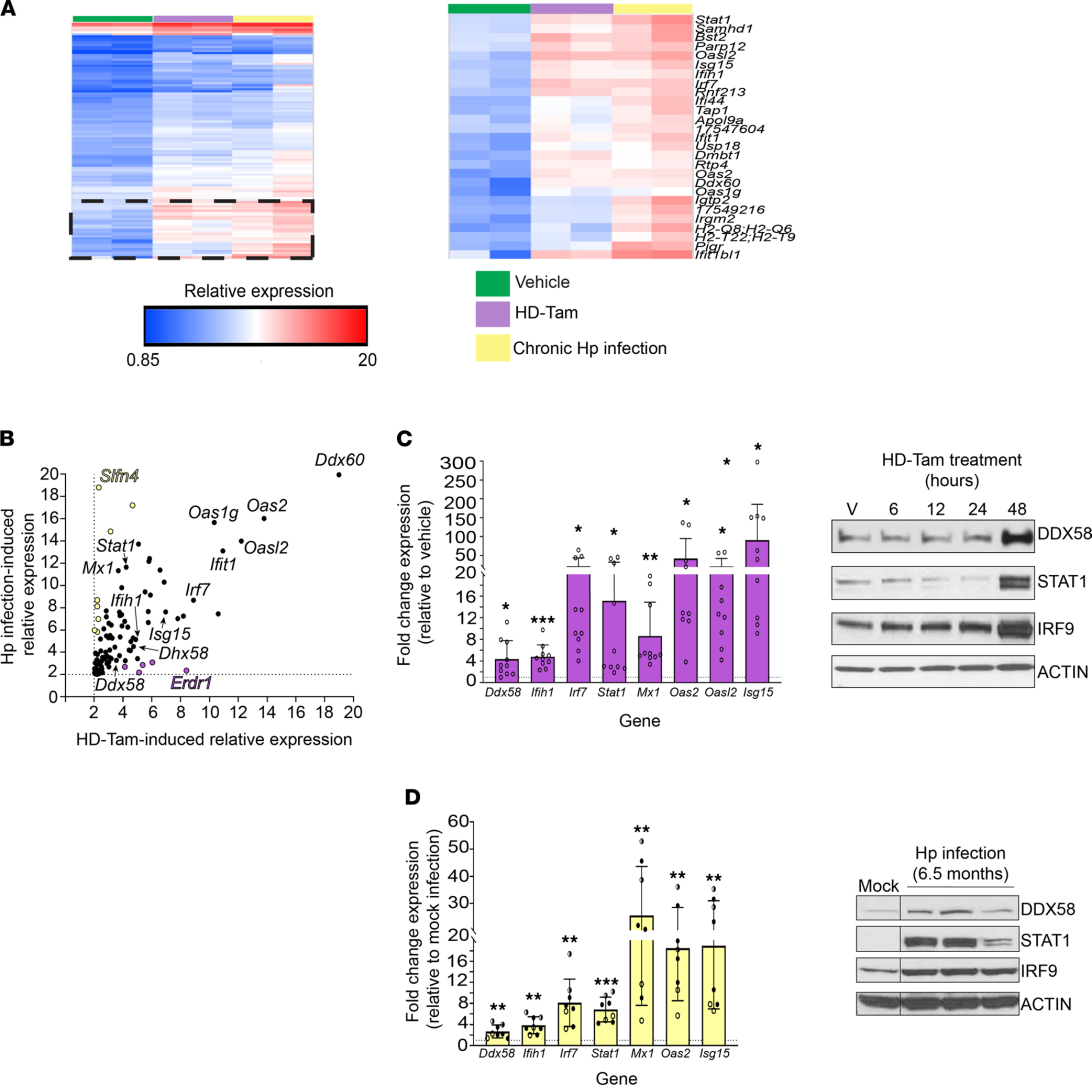
The dsRNA response is upregulated during gastric metaplasia. (**A**) Gene expression profiling of gastric corpus tissue from wild-type mice treated with either vehicle (green) or HD-Tam for 48 hours (purple), or infected with *H*. *pylori* for 6.5 months (yellow). The hatched area is magnified in the right panel, with expression changes for the corresponding genes. (**B**) Genes highlighted in yellow showed higher relative expression following *H*. *pylori* infection, while genes highlighted in purple showed relatively higher expression following HD-Tam treatment. Genes pertaining to the dsRNA response are in black. Genes in yellow and purple have not been shown to be involved in the dsRNA response. Dotted lines represent 2-fold cutoffs. (**C** and **D**) The dsRNA response is activated at the transcriptional (left panels) and protein (right panels) levels following either HD-Tam treatment (**C**) or chronic *H*. *pylori* infection (**D**). For left panels, each data point represents gastric corpus tissue from an individual mouse. Fold expression changes were determined by quantitative reverse transcription PCR (qRT-PCR) and are relative to vehicle-treated (**C**) or mock-infected mice (**D**). The dotted line represents the average expression in vehicle-treated or mock-infected mice. For **C**, pooled data from 3 consecutive, independent experiments are shown. For **C**, “V” corresponds to vehicle treatment. For **D**, each data point represents an individual mouse infected with *H*. *pylori* for 3 months (unfilled circles), 6.5 months (half-filled circles), or 11 months (filled circles). Each lane of the Western blots represents gastric corpus tissue of a mouse from a representative experiment under each experimental treatment condition. For left panels, *P* values were determined using 2-tailed Student’s *t* test, where *, *P* < 0.05; **, *P* < 0.01; ***, *P* < 0.001. HD-Tam, high-dose tamoxifen; Hp, *Helicobacter pylori*.

We next used gene expression profiling to take an unbiased approach in determining whether the response to dsRNA was conserved across these 2 disparate models of gastric metaplasia. Indeed, the most highly upregulated gene pathways across both models of metaplasia were related to innate immune sensing and signaling in response to dsRNA (Figure 2A and Table 2), and many of the most substantially induced transcripts were various components of the dsRNA response. In particular, components of the response pathway that sense dsRNA and amplify downstream signaling (42) were highly represented (Table 2). While certain genes were specific to the individual injury model — e.g., *Slfn4* specific to chronic *H. pylori* infection (43) and *Erdr1* specific to HD-Tam — and not previously shown to our knowledge to be involved in the dsRNA response, a substantial subset of genes related to the dsRNA response was upregulated to a nearly identical extent across both models (Figure 2B). We validated many of the upregulated genes at the protein and mRNA levels in both HD-Tam treatment (Figure 2C) and chronic *H. pylori* infection (Figure 2D). We used commercially available antibodies to localize dsRNA signaling components (e.g., interferon regulatory factor 7, IRF7) within metaplastic glands (Supplemental Figure 3, A–C). In both HD-Tam treatment and *H. pylori* infection, the dsRNA response components were largely detected in chief cells, the same epithelial population in which we had seen an accumulation of dsRNA during the progression to metaplasia (Figure 1A). Downstream components of the dsRNA response, like IRF7, could be found in metaplastic (i.e., basal GSII-positive) cells. As might be expected, the accumulation of dsRNA in chief cells (Figure 1D) preceded the upregulation of the dsRNA response (Supplemental Figure 3C).

### Activation of the dsRNA response during metaplasia is independent of type I IFN signaling.

**Figure 3:**
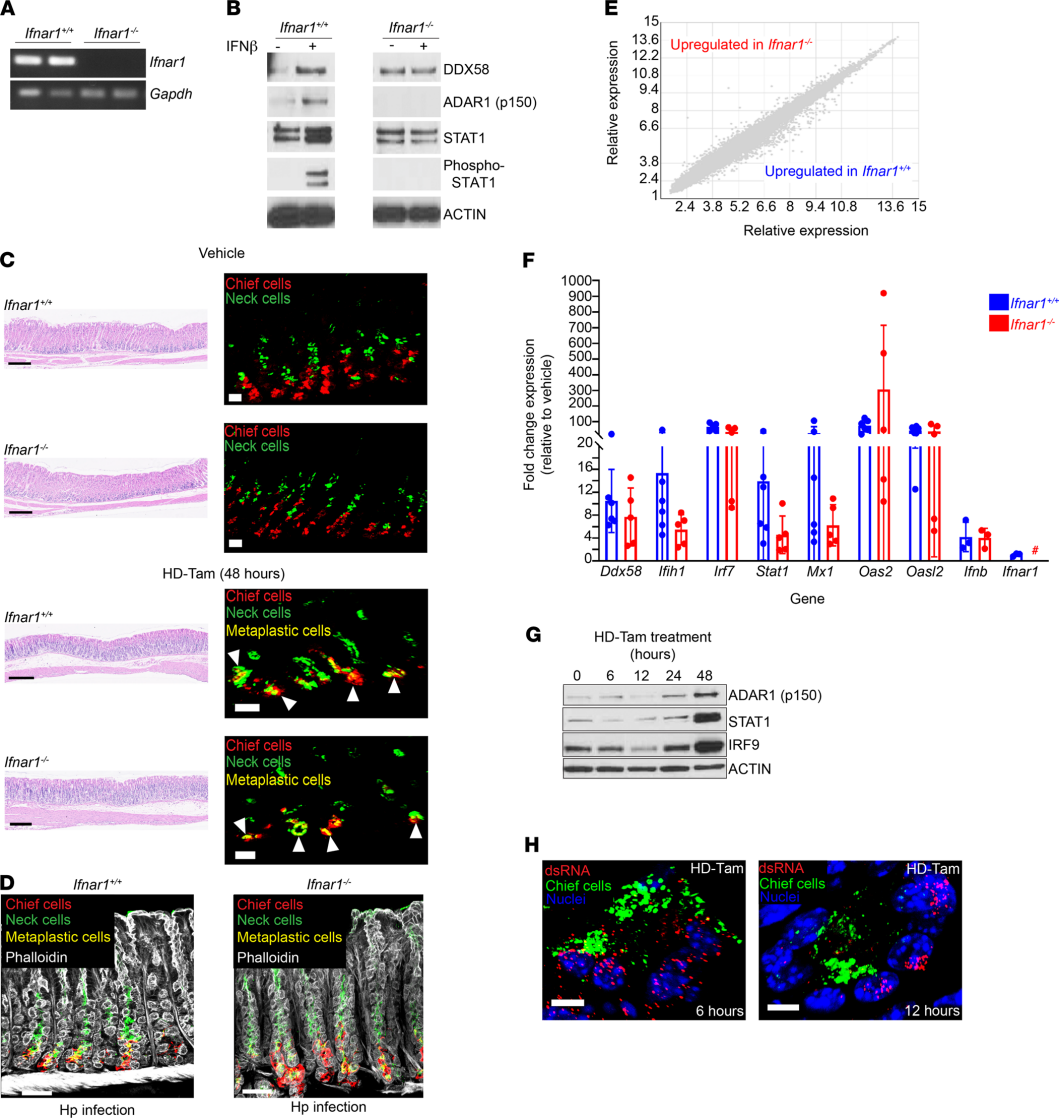
The dsRNA response during gastric metaplasia is independent of IFNAR1 signaling. (**A**) RT-PCR of *Ifnar1* amplicons from gastric corpus tissue for wild-type (*Ifnar1^+/+^*) and age-matched *Ifnar1^–/–^* mice. Each lane represents an individual mouse. *Gapdh*, loading control. (**B**) Gastroids from wild-type or *Ifnar1^–/–^* mice were treated with PBS or IFN-β (100 U/mL) for 24 hours and lysates probed with the indicated antibodies. (**C**) Representative gastric corpus sections of wild-type or *Ifnar1^–/–^* mice treated with vehicle or high-dose tamoxifen (HD-Tam). Right panels highlight representative glands under each treatment. Arrowheads highlight metaplastic glands. Scale bars, 250 μm (left panels), 20 μm (right panels). (**D**) Metaplastic changes in wild-type and *Ifnar1^–/–^* mice infected with *H*. *pylori* (Hp) for 2 weeks. Scale bars, 20 μm. (**C** and **D**) Images are representative of 3 mice per experimental treatment. (**E**) Relative gene expression profiles between wild-type and *Ifnar1^–/–^* mice treated with HD-Tam for 48 hours. Three mice were used per genotype. (**F**) Fold change expression, relative to vehicle-treated, genotype-matched mice, of various dsRNA transcripts was determined by qRT-PCR. Each data point represents gastric corpus tissue from an individual mouse across 2 independent experiments. Means (±SEM) are shown. *P* values were determined using Student’s *t* test. No significant differences (*P* > 0.05) were observed between wild-type and *Ifnar1^–/–^* mice for all the transcripts investigated. #, not detected. (**G**) Gastric corpus tissue from *Ifnar1^–/–^* mice following HD-Tam treatment. Each lane shows a representative mouse from 3 mice per time point. (**H**) Representative confocal images of *Ifnar1^–/–^* gastric corpus gland bases, following the indicated HD-Tam treatment times, show the accumulation of dsRNA (red). Chief cells are highlighted in green. Images are representative of 3 separate mice per time point. Scale bars, 5 μm.

Recognition of dsRNA leads to downstream signaling and the production of type I IFN and ISGs that amplify the cellular response through autocrine and paracrine mechanisms (5, 6). One aspect of this response is the release of type I IFN and engagement of the IFNAR1/2 receptor in a paracrine or autocrine manner. This can lead to propagation of an inflammatory signal via downstream signaling pathways, including STAT1 phosphorylation (6). To test whether gastric metaplasia was dependent on IFNAR1 signaling, we treated *Ifnar1^–/–^* mice with HD-Tam. As expected, *Ifnar1^–/–^* mice lacked *Ifnar1* mRNA (Figure 3A). Additionally, loss of *Ifnar1* was sufficient to abrogate type I IFN signaling as a whole, as gastroids derived from *Ifnar1^–/–^* mice, unlike gastroids from wild-type mice, did not respond to exogenous IFN-β (Figure 3B). Surprisingly, the loss of type I IFN signaling did not cause any detectable defect in the induction of metaplasia (as determined histologically), following either HD-Tam treatment or chronic *H. pylori* infection (Figure 3, C and D). Moreover, the transcriptional response of *Ifnar1^–/–^* mice to HD-Tam was not significantly different from that of wild-type mice (Figure 3E), and, strikingly, the dsRNA response was activated in the stomachs of *Ifnar1*^–/–^ mice to an almost identical extent as wild-type mice at the mRNA and protein levels (Figure 3, F and G). Accordingly, dsRNA accumulated within metaplastic gland bases in the stomachs of *Ifnar1^–/–^* mice (Figure 3H). Together, these results indicate that activation of the dsRNA response and the development of gastric metaplasia do not depend on signaling through the type I IFN receptor.

### Assessing the role of ADAR1, a key regulator of the dsRNA response, within gastric epithelium.

**Figure 4:**
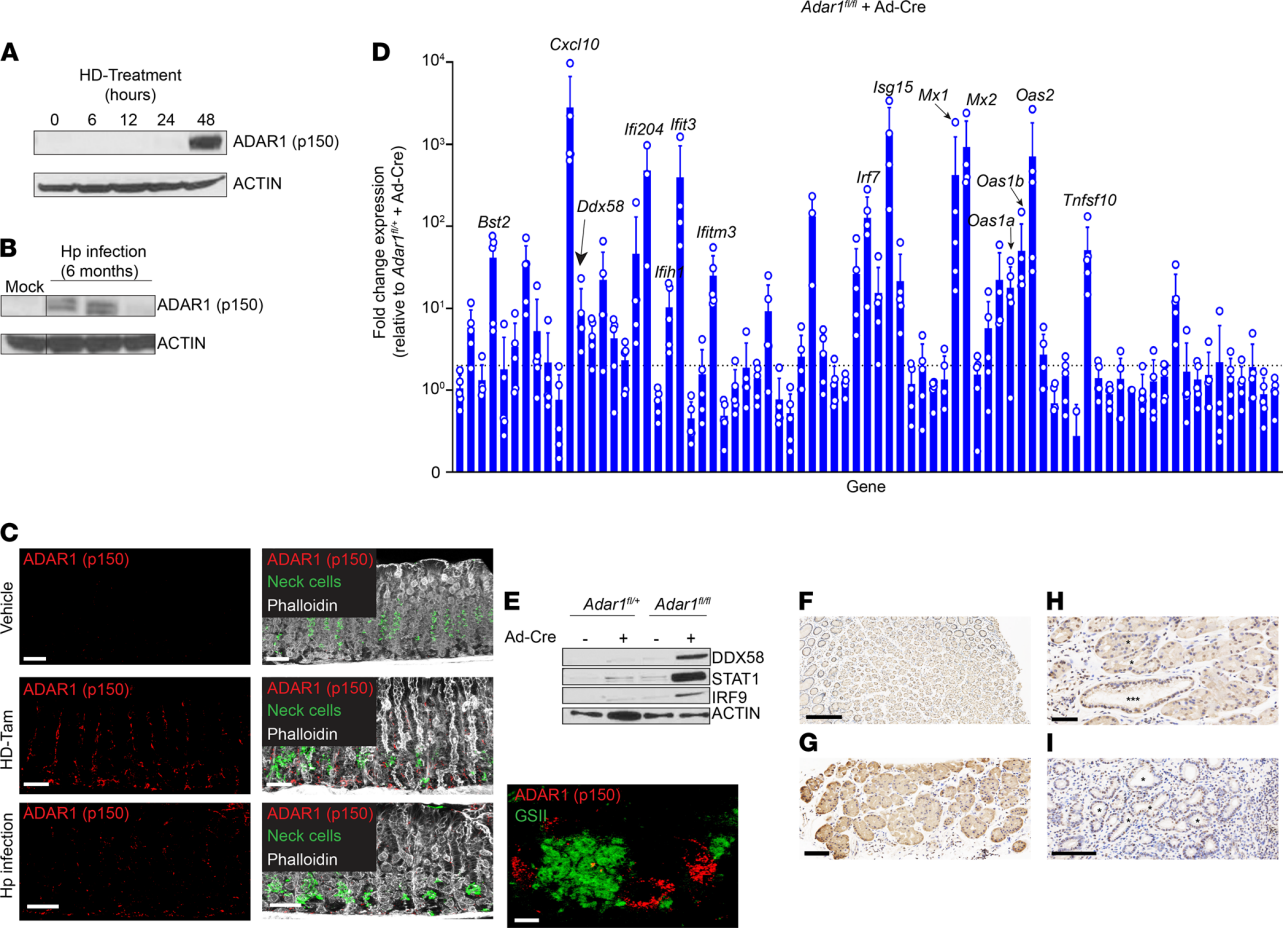
ADAR1 is activated within metaplastic gastric epithelium. (**A** and **B**) The IFN-inducible (p150) isoform of ADAR1 is upregulated in metaplastic corpus tissue following either HD-Tam treatment (**A**) or chronic *H*. *pylori* infection (**B**). Each lane of the Western blot shows gastric corpus tissue from a representative mouse from 3 independent experiments. (**C**) Isolated ADAR1 (p150) staining (left) and merged images (right) are shown. A metaplastic gland base from an *H*. *pylori*–infected mouse stomach with increased cytoplasmic ADAR1 (p150) expression is shown (bottom right panel). Scale bars, 20 μm, and 5 μm (bottom right panel). Neck cells are highlighted by GSII staining (green). (**D**) *Adar1* was deleted from murine gastric epithelium by transducing gastroids from *Adar1^fl/fl^* mice with an adenoviral Cre vector (Ad-Cre). Fold change expression denotes transcript levels, relative to Ad-Cre–transduced gastroids derived from *Adar1^fl/+^* mice, from qRT-PCR using a TaqMan array of dsRNA signaling genes. The means (±SD) from 4–5 independent experiments are shown. Each data point represents gastroid-derived RNA from an individual mouse. Dotted line denotes 2-fold cutoff. (**E**) Representative Western blot of lysates from *Adar1^fl/+^* or *Adar1^fl/fl^* gastroids, either untransduced or transduced with Ad-Cre. (**F**) ADAR1 expression in a representative uninflamed gastric corpus biopsy. (**G**) Representative gastric corpus biopsy from a patient with chronic gastritis and little to no SPEM. Cytoplasmic ADAR1 staining (brown) can be seen in epithelial cells within inflamed gland bases that have not yet progressed to SPEM. (**H**) Representative gastric corpus biopsy from a patient with chronic atrophic gastritis highlighting a hybrid region. Gland bases with early (*) or more advanced SPEM (***) are shown. (**I**) Representative gastric corpus biopsy from a patient with diffuse SPEM. Some of the SPEM gland bases are marked (*). Scale bars, 100 μm (**F** and **I**), 50 μm (**G** and **H**). GSII, *Griffonia simplicifolia* lectin.

Given that the dsRNA response was independent of IFNAR1 signaling, we aimed to identify how the dsRNA response is regulated during metaplasia. The RNA-editing deaminase ADAR1 has been implicated as being central to regulating intracellular responses against both foreign and endogenous dsRNA (15, 44). ADAR1 is ubiquitously expressed in a constitutive, nuclear p110 isoform and can be expressed in an IFN-inducible, activated cytoplasmic p150 isoform (45, 46). Like other components of the dsRNA response, the activated p150 isoform of ADAR1 was upregulated following both HD-Tam treatment and chronic *H. pylori* infection (Figure 4, A and B), and its expression was increased in gastric epithelium undergoing metaplasia (Figure 4C). Moreover, activated ADAR1 expression was significantly upregulated following HD-Tam treatment of germ-free mice, suggesting that ADAR1 activation was a cell-intrinsic consequence of HD-Tam–induced metaplasia and not the result of uptake of viral dsRNA (Supplemental Figure 1D).

Along those lines, to demonstrate ADAR1’s role specifically within gastric epithelium, we derived gastroids from *Adar1*-floxed (*Adar1^fl/fl^*) gastric tissue and transduced them ex vivo with a *Cre* recombinase–expressing adenoviral vector (Ad-Cre). The transduction efficiency of the Ad-Cre, and by extension the efficiency of *Cre*-mediated deletion of *Adar1*, were determined by transducing gastroids derived from *Adar1^fl/fl^* mice bearing a *ROSA26*^LSLTdTomato^ reporter allele. Loss of *Adar1* did not significantly affect gastroid growth (Supplemental Figure 4). Compared with Ad-Cre–transduced *Adar1^fl/+^* gastroids, *Adar1^fl/fl^* gastroids transduced with Ad-Cre showed a robust activation of the dsRNA response at the mRNA (Figure 4D) and protein levels (Figure 4E).

We next examined our series of chronic gastritis specimens from human gastric corpus biopsies described above that were immunostained for total ADAR1. Chief cells from an uninflamed gastric corpus were largely negative for cytoplasmic ADAR1 (Figure 4F). However, in patients with chronic gastritis, cytoplasmic expression of ADAR1, indicative of an increase in the IFN-inducible p150 isoform, could be appreciated in gland base cells maintaining chief cell morphology (Figure 4G). Cytoplasmic ADAR1 expression tended to be weaker in regions where the gland bases had diffusely progressed to an SPEM pattern (Figure 4I; Supplemental Figure 5, C and D; and Table 3). In hybrid regions with early SPEM and transitional morphologies (Figure 4H and Supplemental Figure 5, A and B), cells with more cuboidal, mucinous morphology, consistent with SPEM, tended to have stronger nuclear ADAR1 expression and weaker cytoplasmic ADAR1 expression, with the converse being the case for cells preserving their chief cell morphology. As chronic atrophic gastritis persists in severity, intestinal metaplasia can be seen in regions of SPEM (47). Both intestinal metaplasia and SPEM had scant cytoplasmic and more abundant nuclear ADAR1 expression (Supplemental Figure 5D). Overall, the pattern of expression was consistent with the pattern of dsRNA accumulation observed in inflamed and metaplastic gastric epithelium in murine and human stomachs (Figure 1). Taken together, our findings demonstrate that ADAR1 is activated within mouse and human gastric epithelium undergoing metaplasia and that the dsRNA response can occur in an epithelium-autonomous manner (i.e., independent of immune and/or mesenchymal cell contribution) if one of the enzymes critical to regulating the response to dsRNA, *Adar1*, is deleted.

### Loss of Adar1 from gastric chief cells activates the dsRNA response in vivo.

**Figure 5:**
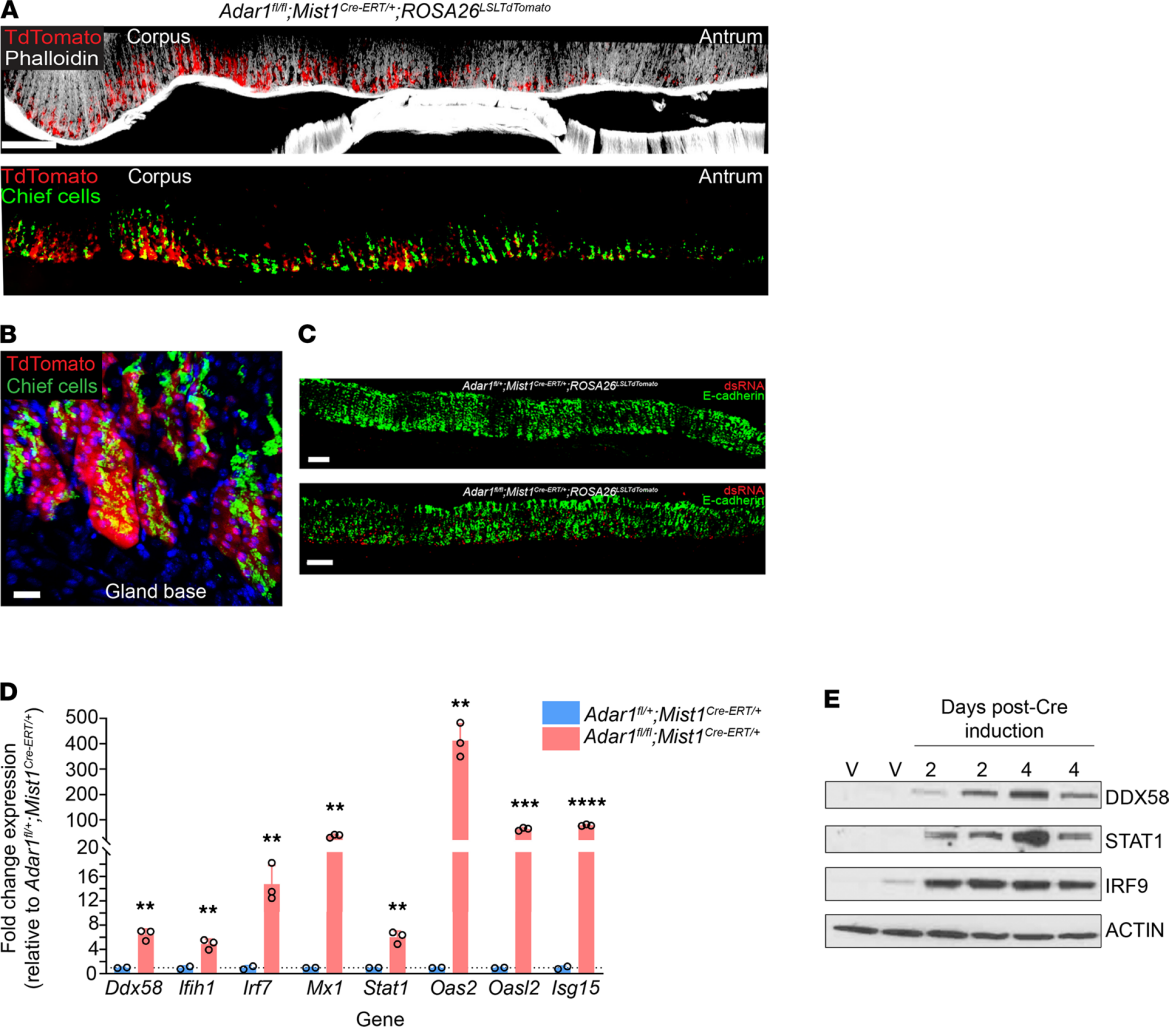
Deletion of *Adar1* from gastric chief cells results in the accumulation of dsRNA and activation of the dsRNA response at homeostasis. (**A**) A representative thick section of gastric tissue from an *Adar1^fl/fl^ Mist1^Cre-ERT/+^ ROSA26^LSLTdTomato^* mouse, 4 days after Cre induction, shows Cre recombination (red; endogenous TdTomato signal) occurring specifically within chief cells (green; gastric intrinsic factor, GIF) of the gastric corpus. (**B**) An isolated corpus gland base is shown, illustrating Cre recombination (red) in chief cells (green). Scale bars, 100 μm (**A**) and 5 μm (**B**). (**C**) Conditional deletion of *Adar1* from chief cells (bottom) results in the accumulation of dsRNA (red) within gastric epithelium (green). No appreciable dsRNA was detected in *Adar1^fl/+^ Mist1^Cre-ERT/+^* gastric epithelium following Cre induction (top). Scale bars, 50 μm. (**D**) Fold expression changes, as determined by qRT-PCR, for various transcripts in *Adar1^fl/fl^ Mist1^Cre-ERT/+^* gastric corpus (pink), relative to *Adar1^fl/+^ Mist1^Cre-ERT/+^* gastric corpus (blue), are shown. Gastric corpus tissue was collected 4 days after the completion of Cre induction. Each data point represents an individual mouse. The dotted line represents the average fold change in *Adar1^fl/+^ Mist1^Cre-ERT/+^* mice. *P* values were determined by 2-tailed Student’s *t* test: **, *P* < 0.01; ***, *P* < 0.001; ****, *P* < 0.0001. (**E**) Representative Western blot demonstrates the activation of various components of the dsRNA response following Cre-mediated deletion of *Adar1*. Each lane represents gastric corpus tissue from an individual *Adar1^fl/fl^ Mist1^Cre-ERT/+^* cage mate/littermate mouse at the indicated time point following the completion of Cre induction. “V” refers to vehicle-treated mice, 4 days after the completion of Cre induction, suggesting that any potential leaky Cre expression does not result in activation of the dsRNA response.

While loss of *Adar1* from gastroids activated the dsRNA response ex vivo, we wanted to examine the effects of *Adar1* deletion on the dsRNA response specifically within chief cells in vivo (48, 49). We crossed our *Adar1*-floxed reporter mice (*Adar1^fl/fl^ ROSA26^LSLTdTomato^*) to mice expressing a tamoxifen-inducible *Cre* under a chief cell–specific promoter (*Mist1^Cre-ERT/+^*; ref. 50). In these mice (*Adar1^fl/fl^ Mist1^Cre-ERT/+^ ROSA26^LSLTdTomato^*), reporter expression was confined to the bases of gastric corpus glands and colocalized with the murine chief cell-specific marker, GIF (Figure 5, A and B), validating specific Cre-mediated deletion of *Adar1* from chief cells. More importantly, loss of *Adar1* from chief cells led to an accumulation of dsRNA within gastric epithelium (Figure 5C, bottom panel). Accordingly, multiple components of the dsRNA response were induced at the transcriptional (Figure 5D) and protein levels (Figure 5E). It is worth noting that the dsRNA response in these mice peaked within 4 days of completing *Cre* induction (see Methods) and subsequently returned to baseline levels by 8 days postinduction (Supplemental Figure 6, A and B). Thus, specifically deleting *Adar1* from gastric chief cells in vivo is sufficient to activate the dsRNA response, in the absence of exogenous gastric injury (i.e., HD-Tam treatment or chronic *H. pylori* infection).

### Loss of Adar1 does not prevent metaplastic gene expression in chief cells.

Given that the dsRNA response, as well as expression of a crucial regulator of this pathway (i.e., ADAR1), peak in cells undergoing metaplasia, we hypothesized that ADAR1 may be playing a key role in metaplastic reprogramming of these cells. Previous studies have shown that this reprogramming proceeds by a stepwise, orderly series of intracellular events, termed paligenosis (25). After initial clearing of cellular components, chief cells undergoing paligenosis first begin to express metaplastic markers (like the epitope for GSII) and subsequently reenter the cell cycle (26). Given the accumulation of dsRNA within metaplastic chief cells (Figure 1A) and our ability to conditionally activate the dsRNA response in this cell population (Figure 5), we wanted to determine how the ADAR1-dependent regulation of the dsRNA response affected paligenosis.

**Figure 6:**
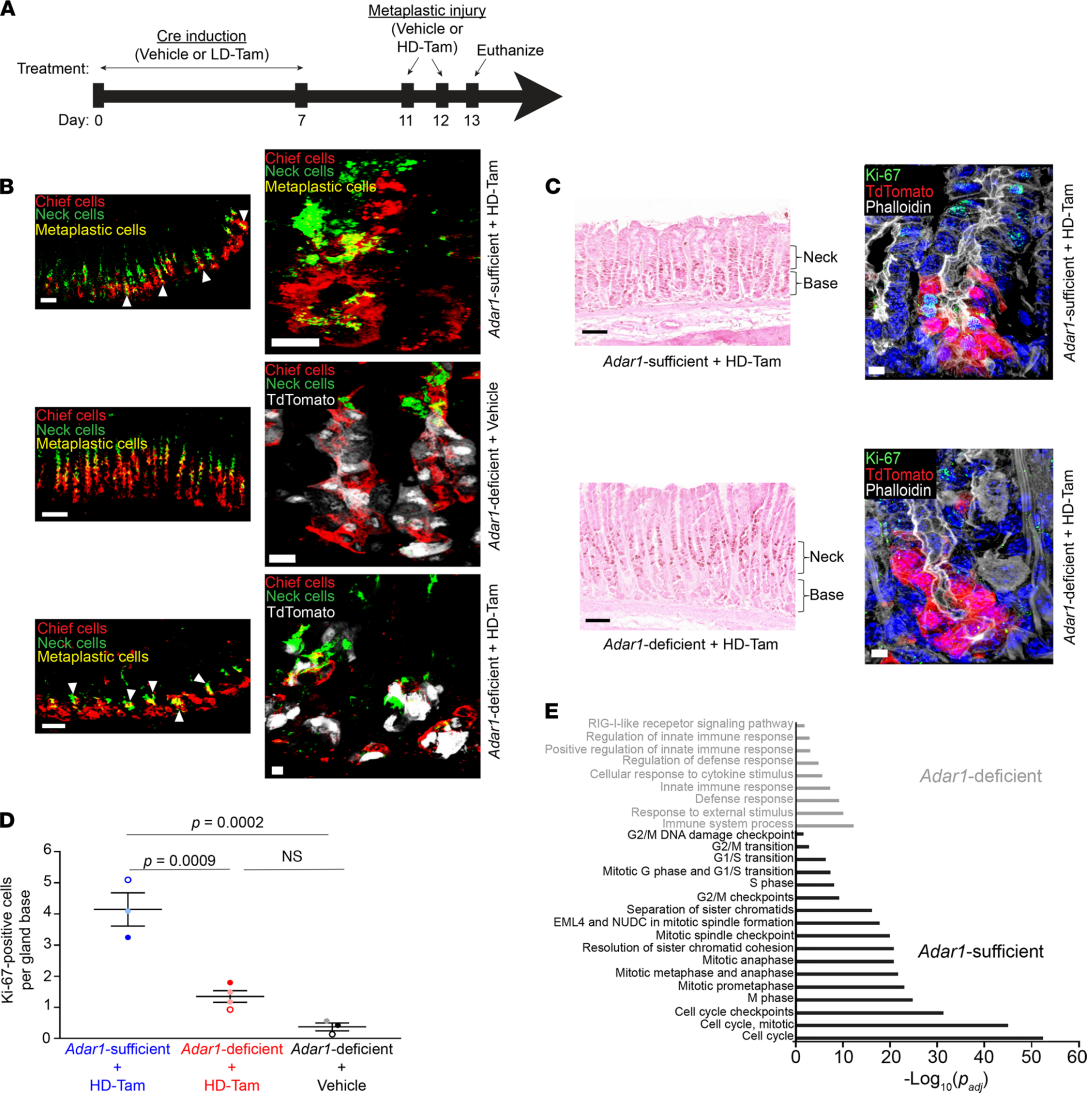
Loss of *Adar1* from chief cells does not affect metaplastic gene expression but limits cellular proliferation following HD-Tam treatment. (**A**) *Adar1^fl/fl^ Mist1^Cre-ERT/+^ ROSA26^LSLTdTomato^* mice were treated with either vehicle or low-dose tamoxifen (LD-Tam) for 7 days to induce Cre-mediated deletion of *Adar1* from chief cells. Four days later, mice were treated with either vehicle or high-dose tamoxifen (HD-Tam) for an additional 2 days to induce metaplasia, then euthanized 1 day after the last injection. (**B**) Metaplastic changes in mice with *Adar1*-sufficient (middle) and -deficient (bottom) chief cells after HD-Tam injury. Arrowheads point to metaplastic glands. Chief cells are labeled with GIF (red), neck cells with GSII (green). Scale bars, 50 μm (left panels), 10 μm (right panels). (**C**) *Adar1*-deficient chief cells show decreased Ki-67 staining following HD-Tam treatment (bottom), compared with HD-Tam–treated, *Adar1*-sufficient chief cells (top). The gland base and neck regions are indicated by brackets. For **B** and **C**, representative gland bases are shown in right panels, with TdTomato signal demonstrating *Mist1^Cre-ERT^* lineage tracing in chief cells. For **B** and **C**, images are representative of 3–4 mice per experimental condition. (**D**) The number of Ki-67–positive cells per gland base was quantified for each experimental setup. Each data point represents the mean number of Ki-67–positive cells per gland across randomly selected fields from an individual mouse, and the mean (±SD) of those data points is indicated. *P* values were determined by 1-way ANOVA using Tukey’s multiple comparisons test. (**E**) Gene set enrichment analysis demonstrates molecular pathways enriched in gastric corpus tissue from *Adar1*-sufficient (black) and -deficient (gray) chief cells after 48 hours of HD-Tam injury. Adjusted *P* values were determined using g:Profiler (68). Microarray data were obtained from 3 individual mice per experimental condition. GIF, gastric intrinsic factor; GSII, *Griffonia simplicifolia* lectin.

We assessed the expression of metaplastic and proliferative markers in metaplastic chief cells in our *Adar1^fl/fl^ Mist1^Cre-ERT/+^ ROSA26^LSLTdTomato^* reporter mice, where we could conditionally and specifically delete *Adar1* from chief cells prior to inducing metaplasia. We triggered *Cre*-mediated deletion of *Adar1* from chief cells prior to metaplastic injury with HD-Tam (Figure 6A). While it has been shown that lower doses of tamoxifen can induce an acute damage response (51), we found that the dose of tamoxifen used to activate Cre recombination in our experimental system (see Methods) did not result in metaplastic changes (Supplemental Figure 6, C and D; and Figure 6B, *Adar1*-deficient + Vehicle). In addition, this low dose of tamoxifen did not lead to an accumulation of dsRNA or transcriptional activation of the dsRNA response in wild-type mice (Supplemental Figure 6, E and F). We observed that metaplastic gene expression was independent of *Adar1*, as HD-Tam–treated stomachs with *Adar1*-deficient chief cells (Figure 6B, bottom panel) demonstrated a pattern of metaplastic marker expression similar to stomachs with *Adar1*-sufficient chief cells (Figure 6B, top panel) following HD-Tam injury.

**Figure 7:**
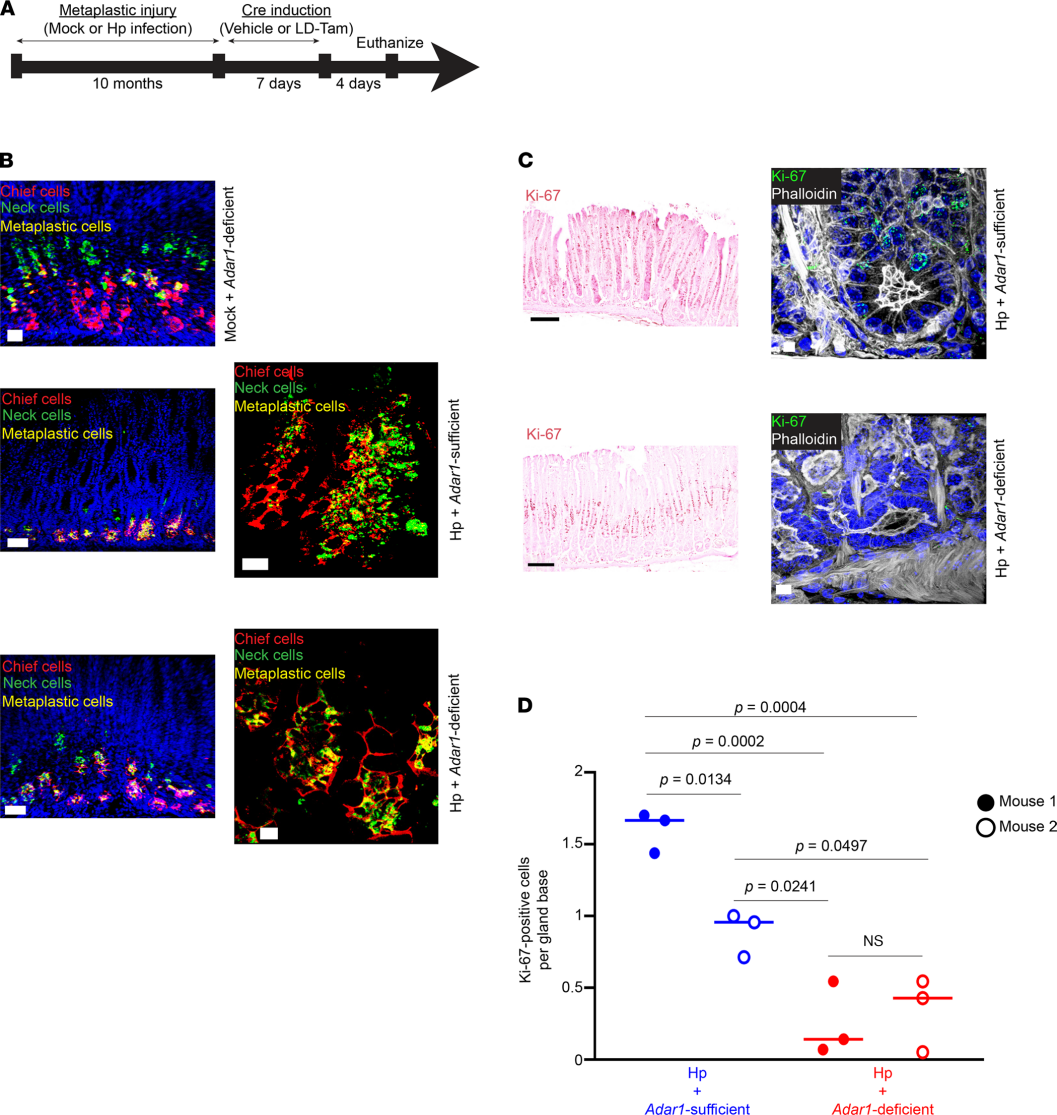
Loss of chief cell–specific *Adar1* does not affect metaplastic gene expression but limits proliferation during chronic *H. pylori* infection. (**A**) *Adar1^fl/fl^ Mist1^Cre-ERT/+^* mice were either mock-infected or infected with *H*. *pylori* (Hp) for 10 months to induce metaplasia, followed by 7 days of either vehicle or low-dose tamoxifen (LD-Tam) treatment to induce Cre-mediated deletion of *Adar1* from chief cells. Mice were euthanized 4 days later. (**B**) During chronic *H*. *pylori* infection, *Adar1*-deficient chief cells (bottom) acquire metaplastic changes similar to chronically infected *Adar1*-sufficient chief cells (middle). Representative metaplastic gland bases (right panels) are shown. Scale bars, 50 μm (left panels), 10 μm (right panels). (**C**) *Adar1*-deficient chief cells show decreased Ki-67 staining following *H*. *pylori* infection (bottom), compared with chronically infected *Adar1*-sufficient chief cells (top). Representative gland bases (right panels) are shown. Scale bars, 50 μm (left panels), 5 μm (right panels). For **B** and **C**, images are representative of 2 mice per experimental treatment. (**D**) The distributions of Ki-67–positive cells at the gland base following *H*. *pylori* infection of mice with *Adar1*-sufficient (blue) or -deficient (red) chief cells are shown. Each data point represents a randomly selected field. No significant difference was seen between the distributions of *H*. *pylori*–infected mice with *Adar1*-deficient chief cells, but all *H*. *pylori*–infected mice with *Adar1*-deficient chief cells had significantly fewer Ki-67–positive cells at the gland base compared with *H*. *pylori*–infected mice with *Adar1*-sufficient chief cells. Few, if any, Ki-67–positive chief cells could be appreciated in mock-infected mice with *Adar1*-deficient chief cells, and this number was not determined. *P* values were calculated by 1-way ANOVA using Tukey’s multiple comparisons test. A statistically significant difference (*P* = 0.0022) was also found between *H*. *pylori*-infected, *Adar1*-sufficient and -deficient mice by Mann-Whitney test.

To further corroborate these findings, we used our *Adar1^fl/fl^ Mist1^Cre-ERT/+^* mouse model to conditionally delete *Adar1* from chief cells using low-dose tamoxifen, after inducing metaplasia through chronic *H. pylori* infection, instead of with HD-Tam (Figure 7A). In the absence of infection, the loss of *Adar1* did not cause metaplasia, consistent with experiments in noninjured mice described above (Figure 7B, Mock + *Adar1*-deficient). In addition, as after HD-Tam treatment, chronic *H. pylori* infection caused chief cells to express metaplastic markers, whether they were *Adar1* deficient or sufficient (Figure 7B, middle and bottom panels). Taken together, our results demonstrate that the induction of metaplastic markers during chief cell paligenosis does not require *Adar1*.

### Adar1 licenses chief cells to proliferate during paligenosis.

During paligenosis, injured chief cells reexpress metaplastic markers prior to reentering the cell cycle and undergoing proliferation (28). Though *Adar1* did not appear to affect metaplastic gene expression, we wanted to examine whether *Adar1* regulated the induction of metaplastic proliferation. We used the same experimental systems for conditionally deleting *Adar1* from metaplastic chief cells (Figure 6A and Figure 7A) and assessed cell cycle reentry by staining for Ki-67, a marker for cells in all stages of the cell cycle. Metaplastic injury, induced with either HD-Tam treatment (Figure 6C, top panels) or chronic *H. pylori* infection (Figure 7C, top panels), resulted in the expected increase in cellular proliferation along the gland axis in both stem/progenitor cells higher in the gland as well as within *Adar1*-sufficient, paligenotic chief cells, consistent with previous literature (26, 52). However, conditional deletion of *Adar1* from chief cells dramatically reduced the census of proliferating, paligenotic chief cells following either HD-Tam–induced (Figure 6C, bottom panels) or *H. pylori* infection–induced (Figure 7C, bottom panels) metaplasia. When *Adar1* was deleted from chief cells, the basal contribution to proliferation in the epithelium was specifically abrogated (Figure 6D and Figure 7D). Because *H. pylori*–induced metaplasia, unlike HD-Tam–induced metaplasia, is multifocal and asynchronous, we chose to examine the distribution across mice for each treatment. Accordingly, we saw some variation in the pattern of Ki-67 staining between the 2 *H. pylori*–infected, *Adar1*-sufficient mice (Hp + Vehicle, Figure 7D). However, the distribution of Ki-67 staining patterns in multiple quantified regions between the 2 *Adar1*-deficient mice was not significantly different, and *H. pylori*-induced proliferation was significantly suppressed in all fields of both *Adar1*-deficient mice (Hp + LD-Tam, Figure 7D). The proliferation defect was confined to the basal (paligenotic) cells, as proliferation of progenitor cells higher up in the metaplastic gland axis was not significantly altered by the loss of *Adar1* (Supplemental Figure 7). In addition, pathway analyses of microarray data confirmed that loss of *Adar1* from chief cells prevented the upregulation of multiple cell cycle pathways in response to HD-Tam (Figure 6E). These results therefore demonstrate that *Adar1* is required for chief cells to appropriately engage the proliferative stage during paligenosis.

### Adar1 deficiency promotes apoptosis following metaplastic injury.

**Figure 8:**
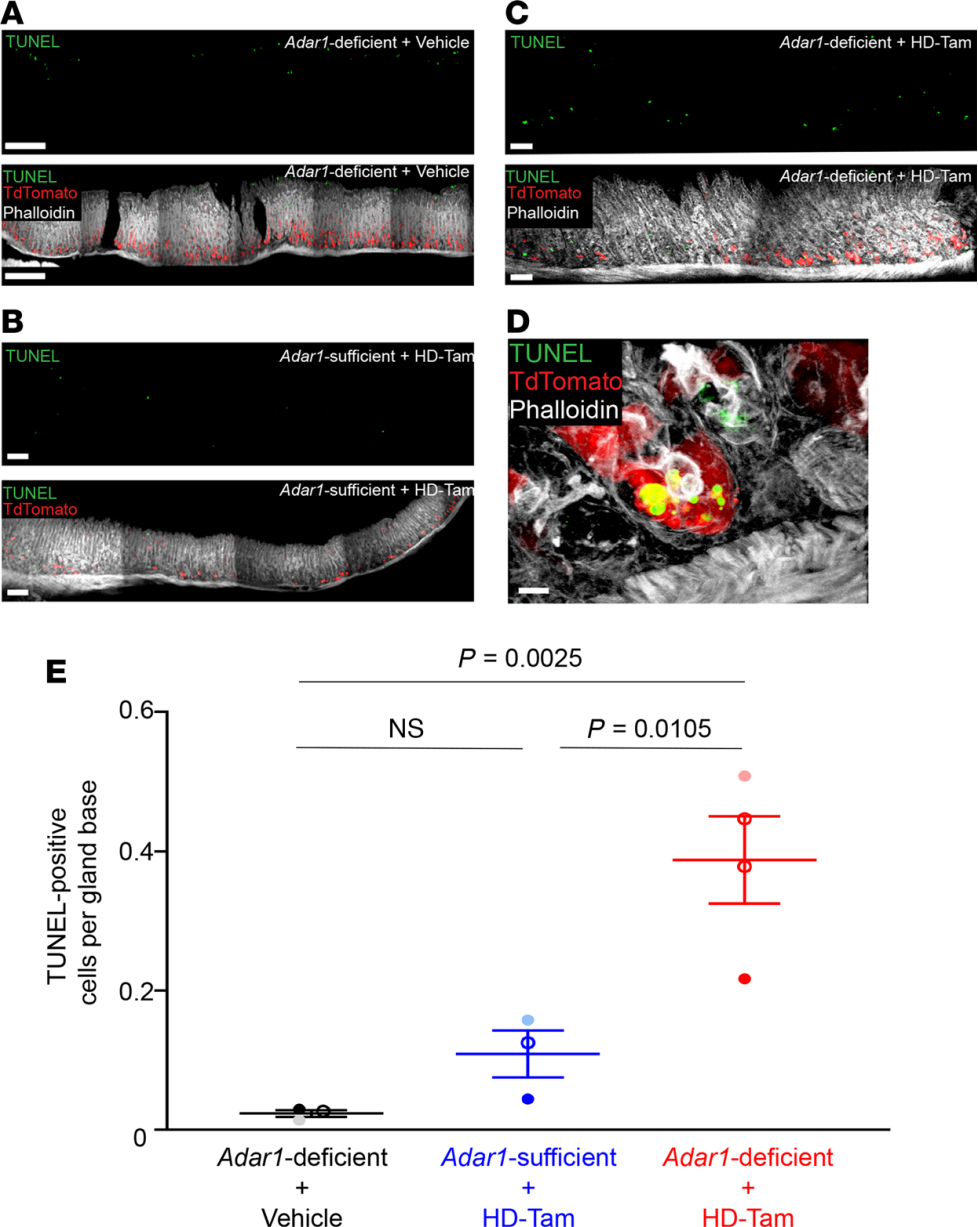
Loss of *Adar1* from chief cells results in more cell death during gastric metaplasia. (**A**–**C**) The loss of *Adar1* from chief cells leads to a greater frequency of dying cells (green) following HD-Tam–induced metaplasia (**C**), compared with HD-Tam–treated stomachs with *Adar1*-sufficient chief cells (**B**). The experimental setup is as shown in Figure 6A. Without HD-Tam injury, chief cell *Adar1* deficiency does not induce cell death at gland bases (**A**). For **A**–**C**, isolated TUNEL signals are shown in the top panels, with merged images in the bottom panels. (**D**) *Adar1*-deficient chief cell (red) undergoing death following metaplastic injury. Scale bars, 50 μm (**A**–**C**), 5 μm (**D**). (**E**) Each data point represents the mean (±SEM) TUNEL-positive cells per gland base from multiple randomly selected fields from an individual mouse. Three to 4 independent experiments are shown. *P* values were determined by 1-way ANOVA using Tukey’s multiple comparisons test.

Previous reports have indicated that ADAR1 regulates epithelial cell survival at homeostasis (53, 54). Though deletion of *Adar1* from chief cells triggered dsRNA signaling (Figure 5, D and E), it did not cause chief cell loss at homeostasis (Supplemental Figure 6D). However, we reasoned that the inability of *Adar1*-deficient chief cells to proliferate during paligenosis could be because accumulation of and/or response to dsRNA during paligenosis is critical for cell survival in a manner not observed during homeostasis. Accordingly, while *Adar1*-deficient chief cells in the absence of metaplastic injury did not undergo any appreciable cell death (Figure 8A, *Adar1*-deficient + vehicle), metaplastic glands containing *Adar1*-deficient chief cells (Figure 8, C–E) showed a higher proportion of TUNEL-positive cells at their bases compared with metaplastic glands with *Adar1*-sufficient chief cells (Figure 8B). It is worth noting that we quantified TUNEL-positive cells only within gland bases, as luminal pit cells undergo apoptosis at homeostasis as part of normal cell turnover (55). Indeed, TUNEL positivity within the pit region served as an internal staining control for our assay (Figure 8A). These results suggest that *Adar1* regulates chief cell survival during paligenosis (but not during homeostasis) and that the loss of *Adar1* from metaplastic chief cells promotes cell death.

## Discussion

Our findings reveal a potentially novel, cell-intrinsic role for ADAR1 and dsRNA signaling in gastric metaplasia, a critical preneoplastic stage in the progression to gastric cancer (56). While dsRNA signaling has been indirectly linked to gastric tumorigenesis (57), our study offers new insight into the importance of ADAR1 during cellular stress and how it dictates epithelial cell fate during injury. It seems likely, though it has not been explicitly shown, that the accumulation of dsRNA may somehow be a normal aspect of development and differentiation. *Adar1*-deficient mice are not viable (18, 58), show increased levels of ISGs that reinforce the IFN response (17), and are rescued to attain adulthood only if they concurrently lack an ability to sense cytosolic dsRNA (18). If one considers that the cellular reprogramming of a postmitotic, differentiated cell during metaplasia (i.e., paligenosis) represents a reversion to a more fetal like state (59), then it would stand to reason that sensing of dsRNA and ADAR1-mediated downstream signaling are inherent to this process.

Though ADAR1 has a prosurvival role in other gastrointestinal tissues at homeostasis (53, 54), our results suggest that gastric epithelial ADAR1 is relevant only during cellular stress. The prosurvival, or rather antideath, function of ADAR1 could occur through several recently proposed mechanisms, all within the context of cellular stress. One of the earliest functional characterizations of ADAR1 suggested its role in regulating survival of murine embryonic fibroblasts during serum starvation (58). A more recent study mechanistically detailed the role of ADAR1 in regulating apoptosis in A172 cells, where the phosphorylated p110 isoform inhibited Staufen 1–mediated decay of antiapoptotic mRNAs (60). In human neural progenitor cells, ADAR1 prevents endogenous RNA from triggering translational shutdown by impeding the phosphorylation and activation of PKR (44). Our results are consistent with these phenotypes and expand ADAR1’s role specifically within a metaplastic cell population in vivo.

The step of paligenosis that decides whether metaplastic cells reenter the cell cycle is critical, as it is carefully licensed to prevent cells carrying DNA damage from proliferating (28). It has been suggested that ADAR1 functions as an oncogene in various cancer cell lines (61) and even in a xenograft model of gastric cancer (32). Our findings suggest that ADAR1 may be critical for oncogenesis because it regulates this key reentry of metaplastic cells into the cell cycle. Indeed, the proliferation of metaplastic epithelium serves a reparative, wound-healing function, but in the setting of sustained injury, every reentry into the cell cycle has the potential to enable the accumulation of mutations that can eventually be unmasked when expressed in proliferating cells (62, 63). In addition to playing a role in allowing progression to the proliferative state, ADAR1 might also play a role in minimizing the mutational burden during metaplasia, through its role as an RNA-editing enzyme (33). In any case, ADAR1 clearly plays a unique role in regulating cell decisions during a crucial precancerous state in the stomach.

dsRNA signaling in vertebrates has become synonymous with the generation of an antiviral state, and it has been assumed that this pathway evolved as a method for cells to sense and defend against viral infection (2). One should consider, however, that the dsRNA response (along with the sensing of other nucleic acids) and the production of type I IFNs may represent an even more evolutionarily conserved method (64) to sense intracellular dsRNA (or other nucleic acids) during the cellular stress that accompanies organ development and/or epithelial injury. Future studies will help identify conserved dsRNA signaling mechanisms that underlie development, metaplasia, and regeneration across other cell types and investigate how therapeutic manipulation of this pathway affects the progression to cancer in an organ-specific context.

## Methods

### Mice.

Mice were maintained in a specified germ-free barrier facility under a 12-hour light cycle. All experiments involving germ-free mice were performed at the Gnotobiotic Core Facility at the Washington University in St. Louis School of Medicine. All mice used for experiments were 6–8 weeks of age. Mice had access to food and water ad libitum. Wild-type C57BL/6, *ROSA26^LSLTdTomato^*, and *Ifnar1^–/–^* male and female mice were obtained from the Jackson Laboratory. The generation and validation of *Adar1*-floxed mice (provided by Qingde Wang, University of Pittsburgh Medical Center, Pittsburgh, PA, USA) have been previously described (58), and all other mice used were generated in-house.

### Antibodies.

Information regarding all antibodies used in this study, including the manufacturer and catalog numbers, is provided in Table 4.

### Tamoxifen experiments.

For HD-Tam experiments, mice were intraperitoneally injected with 2 consecutive daily doses of a tamoxifen/ethanol/sunflower seed oil mixture at a dose of 5 mg/20 g body weight, as previously described (34). For experiments involving *Cre* recombinase induction (LD-Tam), mice were intraperitoneally injected daily with a tamoxifen/ethanol/sunflower seed oil mixture at a dose of 1 mg/20 g body weight for 7 consecutive days. This protocol has been shown to induce *Cre* recombinase without causing metaplasia (51).

### H. pylori infection.

Growth of the wild-type PMSS1 strain of *H. pylori* (provided by Rick Peek, Vanderbilt University, Nashville, Tennessee, USA) has been previously described (65). Briefly, mice were orally gavaged with 200 μL of either Brucella broth (Thermo Fisher Scientific) (mock infection) or PMSS1 diluted in Brucella broth (~1 × 10^8^ CFU/mouse) for the indicated times.

### Microarray analyses.

Gastric corpus tissue from wild-type C57BL/6 littermate/cage mate mice was directly placed in RLT buffer containing 2-mercaptoethanol, and RNA was isolated using the RNeasy Mini Kit (QIAGEN), per the manufacturer’s instructions. Gene expression profiling was performed using microarray analysis in collaboration with the Genome Technology Access Core at the Washington University in St. Louis School of Medicine. RNA was amplified using the WT Plus kit (Thermo Fisher Scientific) and hybridized to Agilent 8 × 60 gene chips. All data were analyzed using the Transcriptome Analysis Console software (Thermo Fisher Scientific). Microarray data were uploaded to the National Center for Biotechnology Information’s Gene Expression Omnibus database with the following accession numbers: GSE190508, GSE190509, and GSE190563.

### Mouse gastroid culture.

The growth and passaging of mouse gastroids was adapted from previously established protocols (66, 67). Mouse gastric corpus glands were isolated by gently stripping away gastric corpus mucosa into gland isolation buffer (5.6 mM Na_2_HPO_4_, 8 mM KH_2_PO_4_, 96 mM NaCl, 1.6 mM KCl, 44 mM sucrose, 55 mM d-sorbitol, 0.5 mM DTT) using fine forceps. The mucosa was incubated in chelation buffer (gland isolation buffer, 10 mM EDTA) for 2 hours at 4°C, then washed 7 times with cold wash buffer (gland isolation buffer without DTT, 1% fetal bovine serum) to isolate gastric glands. Supernatants were pooled and pelleted at 150*g* at 4°C for 5 minutes. The gland pellet was resuspended in Matrigel (Corning), and 50 μL was plated into each well of a 24-well plate supplemented with 500 μL of conditioned medium (Advanced DMEM/F12 from Gibco, HEPES from Corning, GlutaMAX from Gibco, N2 supplement, B27 supplement, 1 mM *N*-acetylcysteine, 50 ng/mL EGF, 200 ng/mL FGF10, 10 nM gastrin, 10 μM Y-27632, primocin, 50% Wnt3a-conditioned medium, 10% R-spondin/noggin–conditioned medium) and incubated at 37°C and 5% CO_2_. Gastroids were passaged as previously described (66). All gastroid-related experiments used gastroids after the first passage.

### Adenoviral transduction of mouse gastroids.

Mouse gastroids were grown as described. Conditioned medium was removed, and gastroids were washed 3 times with cold PBS on ice. Gastroids were resuspended in cold PBS by gently disrupting the Matrigel with pipetting, then pelleted at 150*g* for 5 minutes at 4°C. The pellet was resuspended in TrypLE Express (Gibco) for 5 minutes at 37°C and 5% CO_2_, and trypsinization was quenched with quenching buffer (Advanced DMEM/F12, 10% fetal bovine serum). Cells were pelleted at 150*g* for 5 minutes at 4°C and resuspended in warm conditioned medium. The Ad-Cre vector (Vector Biolabs) was diluted in conditioned medium and added to the cell suspension at a multiplicity of infection of 100. This mixture was gently shaken at 30 rpm at 37°C and 5% CO_2_ for 30 minutes, then pelleted at 2152*g* and 37°C for 5 minutes. The pellet was resuspended in Matrigel on ice, plated onto a 24-well plate, and incubated in conditioned medium at 37°C and 5% CO_2_. Gastroids were processed 4–6 days after transduction.

### Processing of mouse gastroids.

For RNA isolation, gastroids were washed 3 times with cold PBS, then resuspended in cold PBS and pelleted at 2152*g* for 5 minutes at 4°C. RLT buffer containing 2-mercaptoethanol was directly added to the gastroid pellet, and RNA was isolated using the RNeasy Mini Kit, per the manufacturer’s instructions. For protein isolation, gastroids were washed and pelleted, and the pellet was sonicated 3 times in RIPA buffer (Pierce) containing a protease inhibitor cocktail (Thermo Fisher Scientific) on ice. Samples were centrifuged at 21,130*g* for 30 minutes at 4°C, and the supernatants were collected. Protein concentrations in the supernatants were determined using the BCA Protein Assay kit (Thermo Fisher Scientific). For immunofluorescence staining of gastroids, gastroids were washed and pelleted, then fixed in 4% paraformaldehyde (PFA) for 15 minutes at 37°C. Gastroids were washed with PBS, then blocked in blocking buffer (PBS, 3% bovine serum albumin, 1% saponin, 1% Triton X-100) for 2 hours at room temperature. Gastroids were then stained with Alexa Fluor-conjugated phalloidin diluted in blocking buffer (1:100) overnight at 4°C. The next day, gastroids were washed with PBS and incubated in PBS with Hoechst 33258 (1:20,000) for 30 minutes at room temperature. Gastroids were washed and mounted with ProLong Gold antifade reagent (Thermo Fisher Scientific) prior to confocal imaging. The Cytation3 imaging multimode reader (BioTek) was used to obtain bright-field and fluorescent images of gastroids, and the Gen5 Data Analysis software (BioTek) was used for image analysis.

### qRT-PCR.

RNA was extracted using the RNeasy Mini Kit, per the manufacturer’s instructions. RNA was converted to cDNA using the PrimeScript cDNA synthesis kit (Takara). qRT-PCR was performed using SYBR Green on the QuantStudio 3 instrument (Applied Biosystems). For qRT-PCR analysis of mouse gastroid RNA, cDNA was added to a custom TaqMan Array 96-well Fast plate (Applied Biosystems) using TaqMan Fast Advanced Master Mix and TaqMan Gene Expression Master Mix, per the manufacturer’s instructions.

### Western blotting.

Mouse gastric corpus tissue was snap-frozen in liquid nitrogen and stored at –80°C until further use. Tissue was thawed in RIPA buffer containing protease inhibitor cocktail on ice, then sonicated 3 times on ice. Lysates were centrifuged at 21,130*g* for 30 minutes at 4°C, and the protein concentrations from the supernatants were determined using the BCA Protein Assay Kit (Pierce). Lysates were diluted in NuPAGE (Thermo Fisher Scientific) loading buffer and NuPAGE sample reducing agent, then loaded onto NuPAGE 12% Bis-Tris gels. Gels were transferred to 0.45 μm nitrocellulose membranes (MilliporeSigma). Membranes were blocked in Tris-buffered saline with 0.1% Tween-20 containing 5% nonfat milk prior to probing with the appropriate antibodies. Blots were developed using SuperSignal West Pico chemiluminescent substrate (Thermo Fisher Scientific). Information related to the antibodies used for Western blotting, including supplier and catalog numbers, is provided in Table 4. See complete unedited blots in the supplemental material.

### Immunofluorescence.

Mouse stomachs were excised, cut open along the lesser curvature, pinned down in 4% PFA, and fixed overnight at 4°C. The next day, longitudinal stomach strips were embedded in 4% low-melting agarose, and 100 μm sections were cut using a vibratome (Leica Biosystems). Sections were blocked for 2 hours in blocking buffer, then incubated overnight at 4°C in the appropriate primary antibody. The following antibodies were used for immunofluorescence: K1 mouse anti-dsRNA (1:70), rabbit anti–E-cadherin (1:100), GSII (1:1000), rabbit anti-GIF (1:10,000), rabbit anti-IRF7 (1:100), phalloidin (1:100), rabbit anti-ADAR1 (p150 isoform; 1:100; Synaptic Systems), rabbit anti–Ki-67 (1:100), and TUNEL staining kit (MilliporeSigma). Additional information related to the antibodies used for immunofluorescence, including supplier and catalog numbers, is provided in Table 4. Sections were washed and incubated in the appropriate secondary antibodies for 2 hours at room temperature. Sections were then incubated in Hoechst 33258 stain (1:20,000) for 30 minutes at room temperature, transferred to a microscope slide, and mounted using ProLong Gold antifade reagent. Images were obtained using the Olympus FV1200 confocal microscope, and *Z*-stacks were reconstructed into 3-dimensional images using Amaris software (Thermo Fisher Scientific).

### Immunohistochemistry.

Paraffin-embedded (5 μm) sections of human gastric specimens were deparaffinized and rehydrated according to routine paraffin processing protocols. Endogenous peroxidase activity was quenched with 1.5% H_2_O_2_ in methanol for 15 minutes at room temperature. Slides were boiled for 10 minutes in 10 mM sodium citrate (pH 6.0), then blocked for 2 hours at room temperature in blocking buffer (see *Immunofluorescence*) in a humidity chamber. Slides were subsequently blocked using the Avidin/Biotin blocking kit (Vector Laboratories), according to the manufacturer’s protocol. For ADAR1 staining, slides were incubated in rabbit anti-ADAR1 (p110 and p150 isoforms; 1:100; Atlas) overnight at 4°C and washed with PBS the next day. For all quantification of Ki-67 staining, paraffin-embedded, murine gastric corpus tissue was processed as above. Tissue was incubated overnight at 4°C in rabbit anti–Ki-67 antibody (1:100; Abcam), then washed the next day in PBS prior to incubating in the corresponding secondary antibodies. For both ADAR1 and Ki-67 staining, signal was amplified using the VectaStain Elite ABC kit (Vector Laboratories) and developed using 3,3′-diaminobenzidine tetrahydrochloride (Thermo Fisher Scientific). Slides were counterstained with eosin or hematoxylin according to routine staining protocols. For all quantification of Ki-67 staining, gastric corpus sections were scored by an observer blinded to the genotype and treatment. The number of Ki-67–positive cells per gland base, where chief cells and metaplastic chief cells reside and which was uniformly determined by the blinded observer for each quantified strip, was determined.

### Histologic scoring.

For Table 3, gastric corpus biopsies were stained with the ADAR1 antibody and counterstained with hematoxylin. Biopsies were evaluated by a blinded pathologist and scored 1–4, with 4 being most severe for gastritis (i.e., degree of chronic inflammatory infiltrate), PC atrophy (degree of PC loss), SPEM (extent of basal replacement of chief cells by mucous cells), and intensity/extent of cytoplasmic ADAR1 staining in chief cells. IM was evaluated from 0 to 2, with 0 referring to no IM detected, 1 meaning focal IM, and 2 meaning extensive IM.

### Statistics.

For qRT-PCR, mRNA expression was determined using the ΔΔCt method with normalization to the housekeeping gene *Gapdh*. All statistical tests and numbers of biological replicates can be found in the figure legends. Where applicable, results are presented as means ± standard deviation (SD) or standard error of the mean (SEM). All quantification was done by an observer who was blinded to the experimental treatments. Comparisons between treatments were made using either 2-tailed Student’s *t* test, Mann-Whitney test, or 1-way ANOVA using Tukey’s multiple comparisons test, where appropriate. *P* values are provided where appropriate and considered significant for *P* < 0.05. Statistical analyses were performed using GraphPad Prism 9.0. Gene set enrichment analyses, including calculation of adjusted *P* values, was performed using g:Profiler software (68).

### Study approval.

All human gastric specimens were obtained through a collaboration between JBS and the Universidad del Cauca (Popayán, Colombia), with approval from the Institutional Review Boards at the Universidad del Cauca and at the Washington University in St. Louis School of Medicine (IRB 201901176). Informed written consent was obtained for use of these samples as approved by the appropriate Institutional Review Board. All experiments involving mice were performed according to protocols approved by the Washington University in St. Louis School of Medicine Animal Studies Committee.

## Author contributions

JBS and NV designed and conducted the experiments. JBS, NV, CJC, and JCM performed data analyses. JBS and JCM wrote the manuscript. JBS, NV, CJC, and JCM provided editorial input.

## Supplementary Material

Supplemental data

## Figures and Tables

**Table 4:**
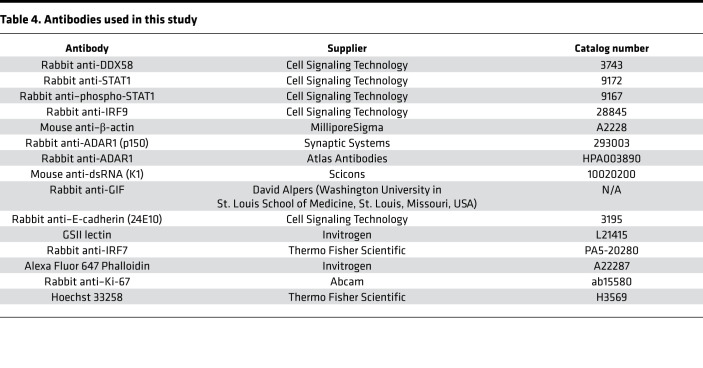
Antibodies used in this study

**Table 1:**
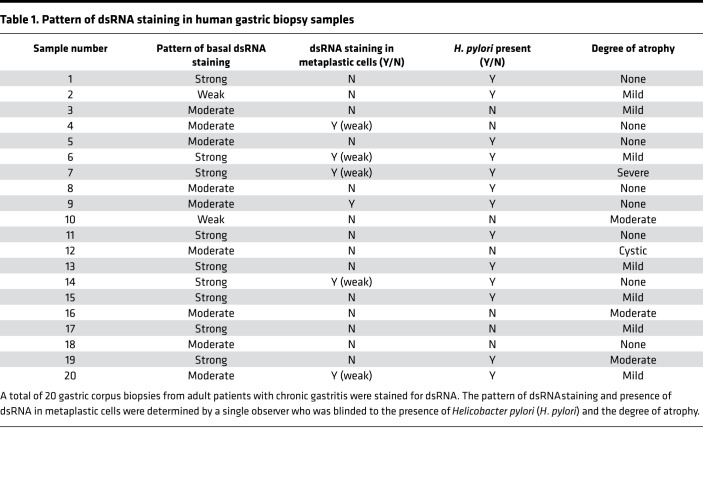
Pattern of dsRNA staining in human gastric biopsy samples

**Table 2:**
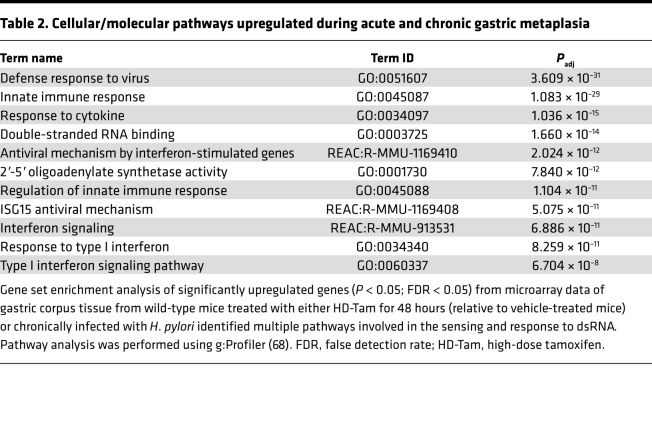
Cellular/molecular pathways upregulated during acute and chronic gastric metaplasia

**Table 3:**
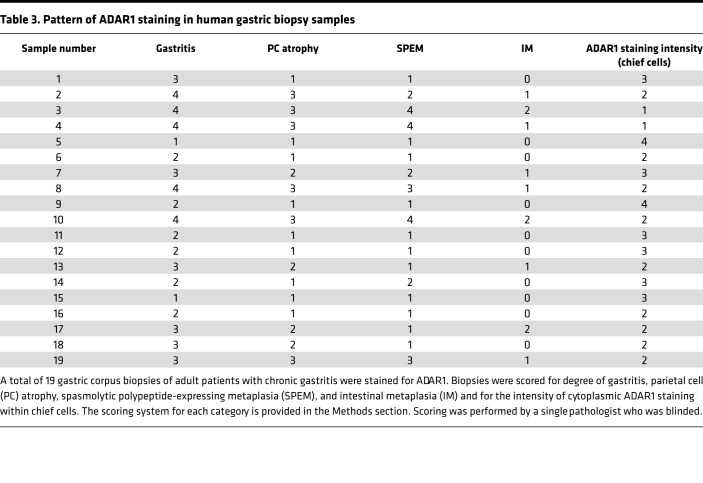
Pattern of ADAR1 staining in human gastric biopsy samples

## Version 1 02/08/2022

Electronic publication
