# Supplementary material for: Regulation of the double-stranded RNA response through ADAR1 licenses metaplastic reprogramming in gastric epithelium
Source: JCI Insight. 2022 Feb 8;7(3):e153511. doi: 10.1172/jci.insight.153511 (PMC8855806; doi:10.1172/jci.insight.153511)

# Supplemental Figure 1

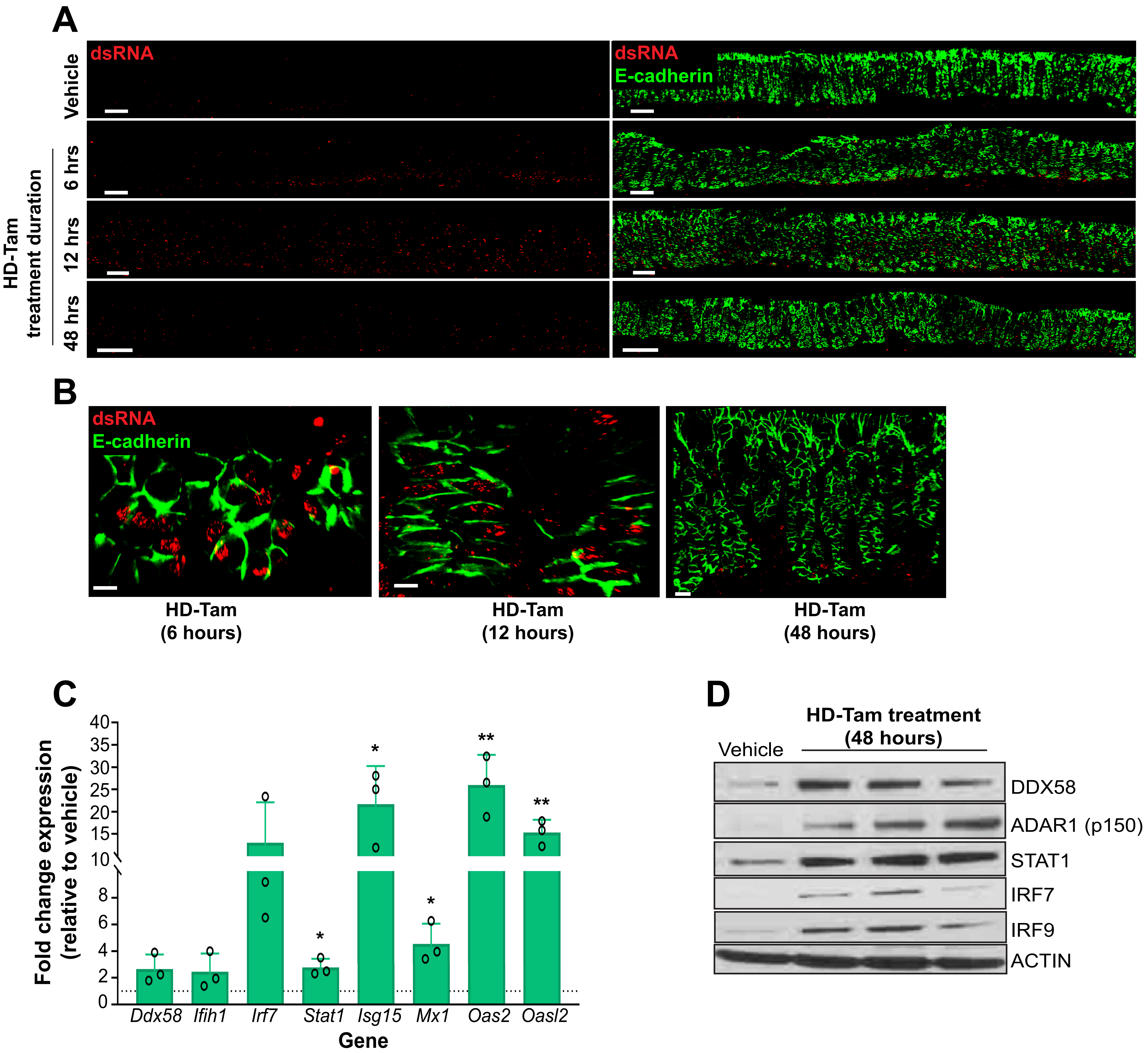


**Supplemental Figure 1. Germ-free mice activate the dsRNA response following HD-Tam treatment.** (**A**) Dynamics of dsRNA accumulation in germ-free (GF) mice. Representative gastric corpus sections of GF mice treated with vehicle for 24 hours or with high-dose tamoxifen (HD-Tam) for the indicated times are shown. Three littermate/cagemate mice were used for each treatment condition The isolated dsRNA signal (red) is shown in left panels. Gastric glands are outlined in green (E-cadherin) and shown in the merged images (right panels). Scale bars, 100 μm. (**B**) High magnification images of gastric gland bases from GF mice treated with HD-Tam at the indicated times. dsRNA (red) accumulates within gastric epithelium (green) at 6 and 12 hours but is largely absent by 48 hours. For 48 hours, entire glands are shown. Scale bars, 10 μm. (**C-D**) Multiple components of the dsRNA response are activated at the transcriptional (C) and protein (D) levels following HD-Tam-induced gastric metaplasia. (C) Fold expression changes (relative to vehicle-treated GF mice) for various dsRNA response transcripts following 48 hours of HD-Tam treatment were determined by qRT-PCR. Each data point represents gastric corpus tissue isolated from an individual littermate/cagemate GF mouse. Means (±S.D.) are shown. The dotted line denotes the average fold change in vehicle-treated mice. *P* values were determined using the Student's *t* test, where *, $p < 0.05$; **, $p < 0.01$. (**D**) HD-Tam treatment activates multiple components of the dsRNA response. Each lane of the representative Western blot shows gastric corpus tissue from an individual, germ-free cagemate/littermate mouse treated for 48 hours with HD-Tam.

## Supplemental Figure 2

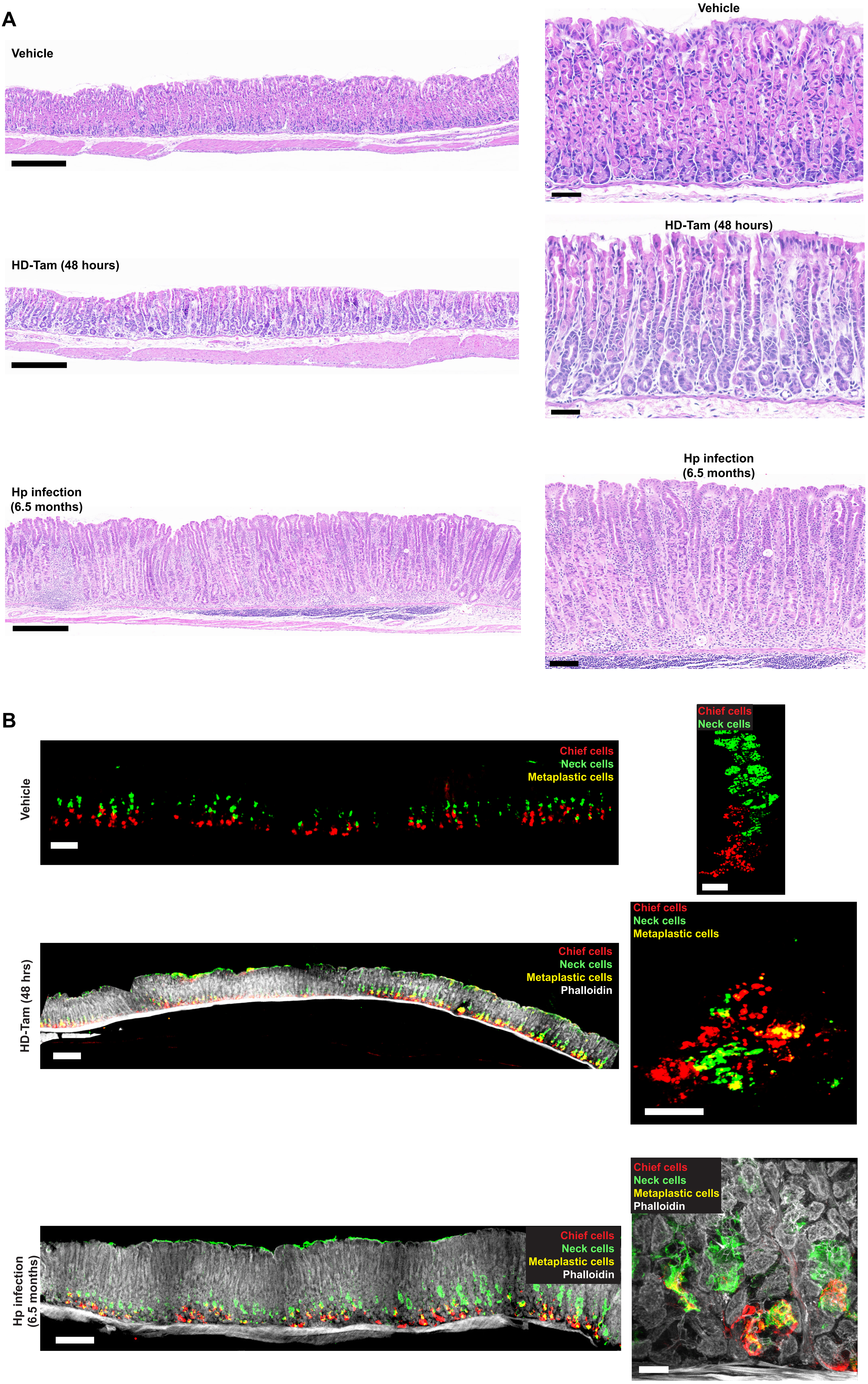


**Supplemental Figure 2. Validation of models of gastric metaplasia.** (**A**) Representative gastric corpus sections of wild-type mice treated with either vehicle, high-dose tamoxifen (HD-Tam) for 48 hours, or infected with *Helicobacter pylori* (Hp infection) for 6.5 months. Right panels highlight magnified images of gastric glands under each treatment condition. For HD-Tam treatment, note the relative lack of inflammatory infiltrate compared to Hp infection. Scale bars, 250 µm (left panels), 20 µm (right panels). (**B**) Both HD-Tam and Hp infection induce histologic changes consistent with gastric metaplasia. Representative gastric corpus sections from HD-Tam-treated and Hp-infected mice (three mice per treatment) demonstrate glandular changes consistent with gastric metaplasia, namely the co-expression of the murine chief cell marker GIF (red) and the mucous neck cell marker GSII (green) at gland bases, compared to vehicle-treated mice. Right panels highlight magnified corpus gland bases for each treatment. Scale bars, 100 µm (left panels), 10 µm (right panels). GIF, gastric intrinsic factor; GSII, *Griffonia simplicifolia* lectin.

# Supplemental Figure 3

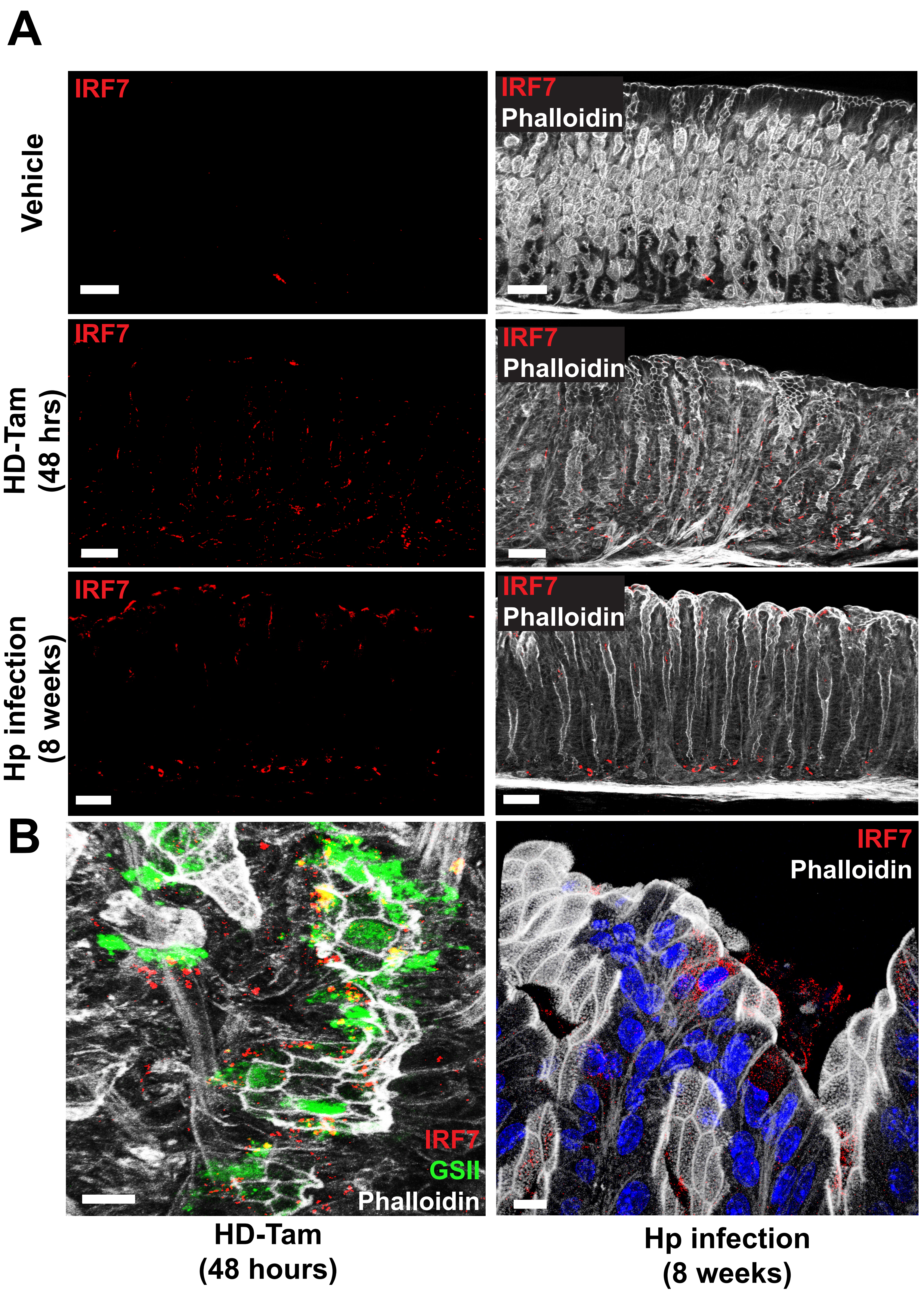


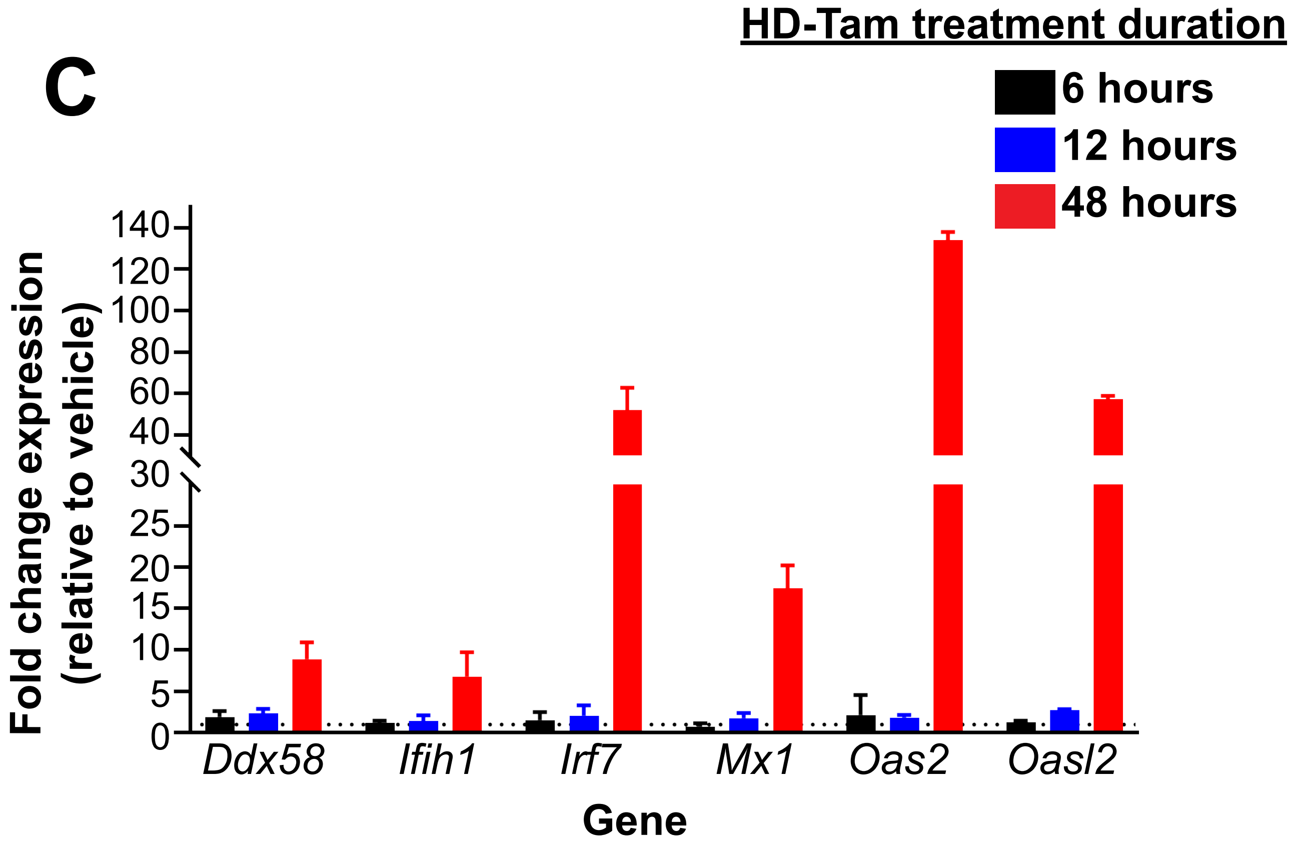


**Supplemental Figure 3. The dsRNA response is activated within gastric epithelium and is time-dependent.** (**A-B**) IRF7 expression is significantly increased during gastric metaplasia. (**A**) Representative gastric corpus sections (three mice per treatment) of wild-type mice treated with either vehicle, high-dose tamoxifen (HD-Tam) for 48 hours, or infected with *Helicobacter pylori* (Hp) for 8 weeks and stained with an antibody to IRF7 (red). Left panels demonstrate the isolated IRF7 signal, and right panels show merged images, with gastric glands highlighted by phalloidin staining (white). While IRF7 is absent from gastric glands in vehicle-treated mice, its expression is significantly increased in HD-Tam-treated and Hp-infected stomachs. (**B**) Representative images showing IRF7 expression at a metaplastic gland base following HD-Tam treatment (left panel), as well as increased expression in the gastric pit of an Hp-infected stomach (right panel). GSII, *Griffonia simplicifolia* lectin (neck cell marker). Scale bars, 50 µm (A) and 5 µm (B). (**C**) Activation of the dsRNA response is time-dependent. Fold expression changes, as determined by qRT-PCR, for various dsRNA response transcripts, relative to vehicle-treated mice, following the indicated HD-Tam treatment times. Bars represent gastric corpus tissue isolated from three individual cagemate/littermate wild-type mice. Means (±S.D.) are shown. The dotted line denotes the average expression in vehicle-treated mice. Note that, for all of the transcripts investigated,expression changes do not significantly increase until after 12 hours of HD-Tam treatment.

## Supplemental Figure 4

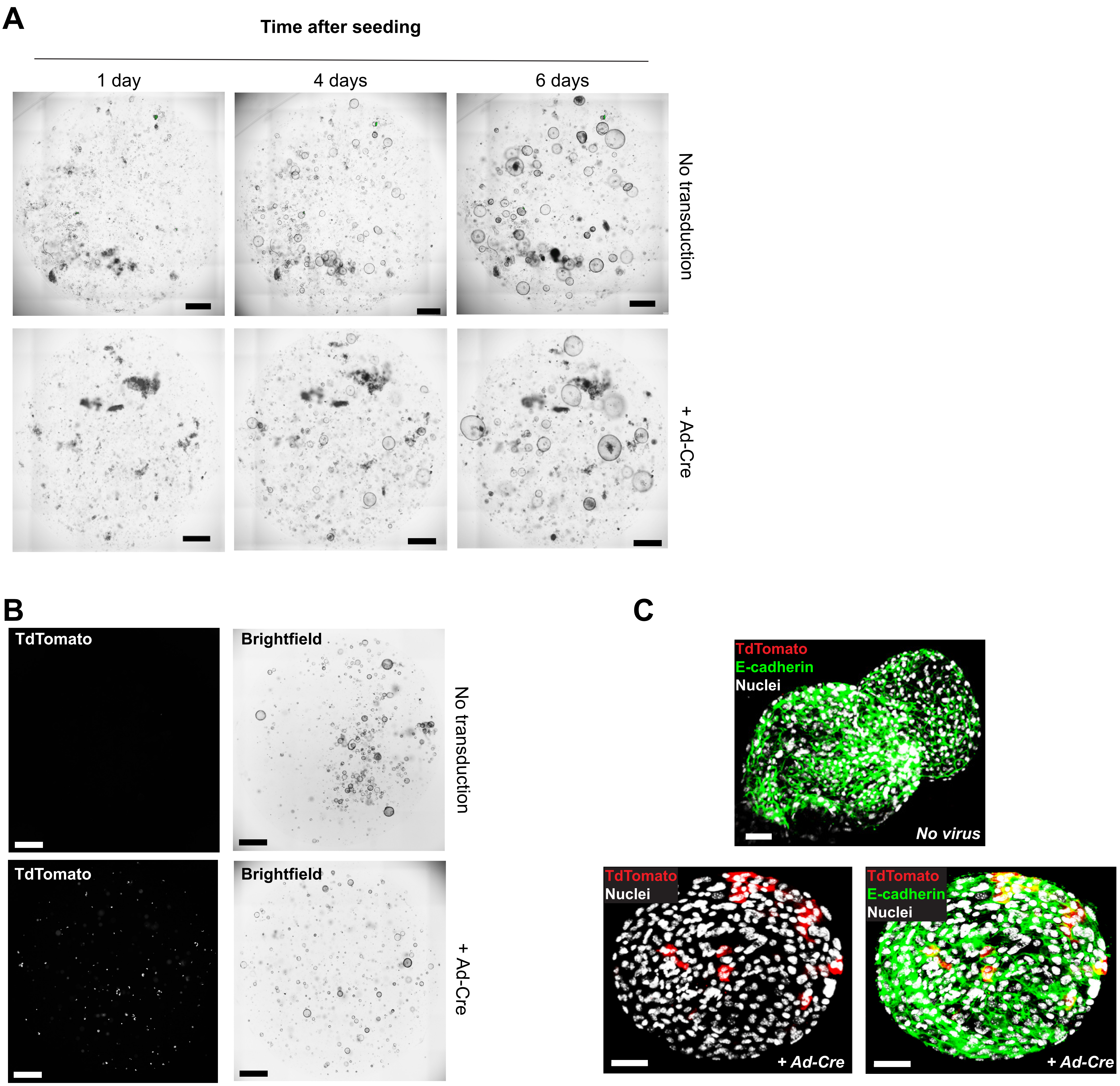


**Supplemental Figure 4. Targeted deletion of *Adar1* from murine gastroids.** (**A**) Gastroids isolated from the gastric corpora of *Adar1$^{fl/fl}$;ROSA26$^{LSLTdTomato}$* mice were either left untransduced (No transduction) or were transduced with an adenoviral-Cre vector (+ Ad-Cre) prior to seeding (see Methods). Representative Brightfield images (of three independent experiments) of gastroids derived from one mouse and taken at the indicated times following transduction are shown. Scale bars, 1 mm. (**B**) Representative images of *Adar1$^{fl/fl}$;ROSA26$^{LSLTdTomato}$* gastroids from one mouse, 4 days after either no transduction or Ad-Cre transduction, are shown. Left panels show the isolated TdTomato signal to indicate the transduction efficiency and the efficiency of Cre-mediated recombination. Right panels demonstrate the corresponding Brightfield images. Scale bars, 1 mm. (**C**) Representative immunofluorescent images of *Adar1$^{fl/fl}$;ROSA26$^{LSLTdTomato}$* gastroids from one mouse, 4 days following either no transduction (No virus) or Ad-Cre transduction (+Ad-Cre), are shown. Transduced cells that underwent Cre-mediated recombination are shown in red. Scale bars, 50 μm.

# Supplemental Figure 5

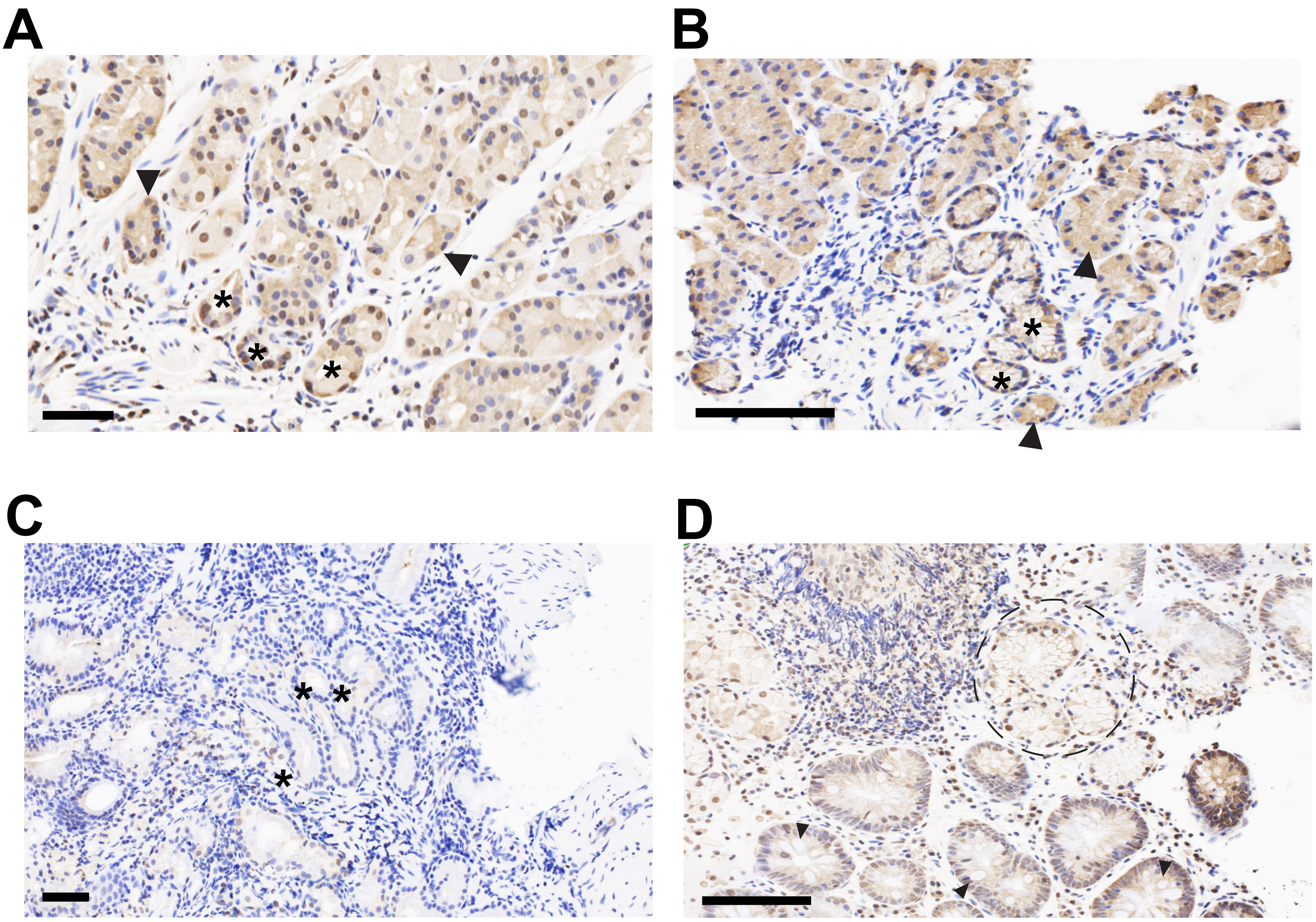


**Supplemental Figure 5. IFN-inducible, cytoplasmic ADAR1 accumulates within inflamed corpus gland bases but is largely absent from SPEM gland bases.** Representative images of gastric corpus biopsies from *Helicobacter pylori*-infected patients. All of the biopsies showed evidence of chronic gastritis. (**A-B**) Cytoplasmic ADAR1, indicative of expression of the IFN-inducible p150 isoform, can be appreciated in inflamed chief cells at gland bases (arrowheads), but cytoplasmic staining is decreased in gland bases with early SPEM changes (*), where ADAR1 nuclear staining, indicative of the constitutive p110 isoform, is more prominent. (**C**) In more advanced SPEM, cytoplasmic ADAR1 staining is decreased or absent. Some SPEM gland bases are marked with an asterisk (*). (**D**) Cytoplasmic ADAR1 staining is decreased in glands exhibiting SPEM or intestinal metaplasia. Gland bases exhibiting SPEM are circled, and glands with intestinal metaplasia are denoted by arrowheads. For panels A and C, scale bars, 50 μm. For panels B and D, scale bars, 100 μm. SPEM, spasmolytic polypeptide-expressing metaplasia.

## Supplemental Figure 6

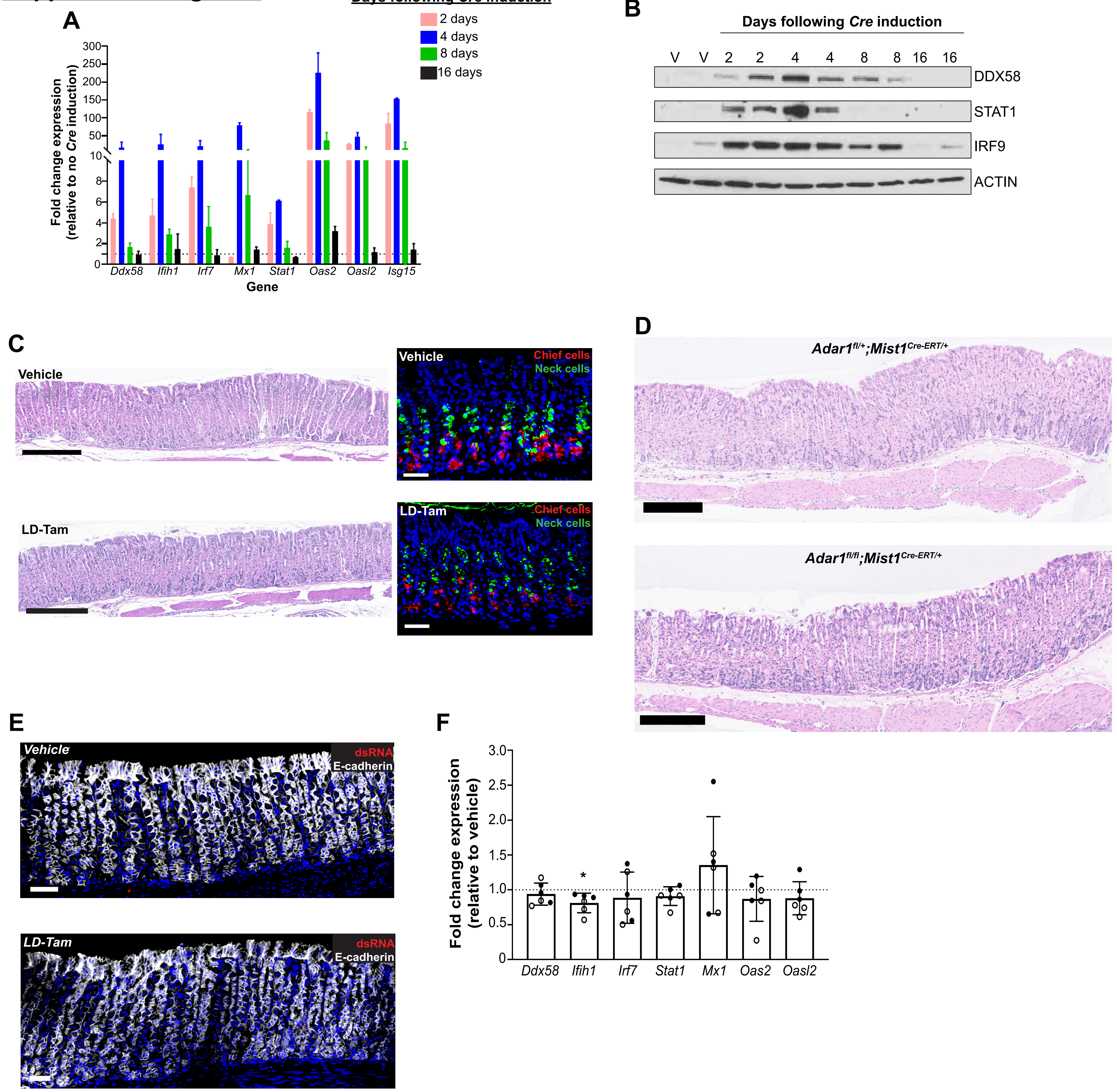


**Supplemental Figure 6. Kinetics of activation of the dsRNA response following *Cre* induction.** (**A-B**) *Adar1*$^{fl/fl}$*;Mist1*$^{Cre\text{-}ERT/+}$ mice were intra-peritoneally injected with LD-Tam for seven consecutive days to induce *Cre* (see Methods). At the indicated time points following the completion of *Cre* induction, gastric corpus tissue was collected, and fold expression changes (relative to no *Cre* induction) for various dsRNA response transcripts were determined by qRT-PCR. Bar graphs represent means (±S.D.) of four individual cagemate/littermate mice per time point. The dotted line denotes average fold change expression in mice without *Cre* induction. (**B**) *Adar1*$^{fl/fl}$*;Mist1*$^{Cre\text{-}ERT/+}$ mice were injected with LD-Tam for seven consecutive days to induce *Cre* (see Methods). At the indicated time points following the completion of *Cre* induction, gastric corpus tissue was collected. A Western blot of tissue lysates is shown. Each lane represents an individual cagemate/littermate mouse at the indicated time point. Note that the peak expression in protein levels of various components of the dsRNA response occurs at 4 days following the end of *Cre* induction.
(**C**) Representative gastric corpus sections of wild-type mice following seven consecutive daily injections with either vehicle (top panels) or LD-Tam (bottom panels). Left panels show gastric corpus tissue stained with hematoxylin/eosin. Right panels show representative confocal images of gastric corpus tissue stained with the chief cell marker, GIF (red), and the mucous neck cell marker, GSII (green). Scale bars, 250 µm (left), 20 µm (right). **(D)** Gastric corpus sections of *Adar1*$^{fl/+}$*;Mist1*$^{Cre\text{-}ERT/+}$ (top) and *Adar1*$^{fl/fl}$*;Mist1*$^{Cre\text{-}ERT/+}$ (bottom) mice stained with hematoxylin/eosin, 4 (top) or 30 (bottom) days following the completion of *Cre* induction. Scale bar, 250 µm. **(E-F)** LD-Tam treatment does not induce an accumulation of dsRNA or transcriptionally activate the dsRNA response. In (E), representative gastric corpus sections from wild-type mice treated with either LD-Tam (top) or vehicle (bottom) and stained for dsRNA (red) are shown. Epithelial cells are outlined in white. Scale bars, 50 µm. (F) Fold expression changes for various dsRNA response transcripts, quantified by RT-PCR, are shown for gastric corpus tissue for LD-Tam-treated, wild-type mice, relative to vehicle-treated, wild-type mice. Each data point represents an individual mouse. Bar graphs represent means (±S.D.). Open circles correspond to female mice, closed circles to male mice. The dotted line denotes the average fold change for vehicle-treated mice. No statistically significant differences were detected between vehicle and LD-Tam treatment by the Student's *t* test, except for *Ifih1*, which was signficantly reduced (*, $p < 0.05$) following LD-Tam treatment. For C-E, all images are representative of six individual mice for LD-Tam treatment and 3 individual mice for vehicle treatment. dsRNA, double-stranded RNA; LD-Tam, low-dose tamoxifen; GIF, gastric intrinsic factor; GSII, *Griffonia simplicifolia* lectin.

# Supplemental Figure 7

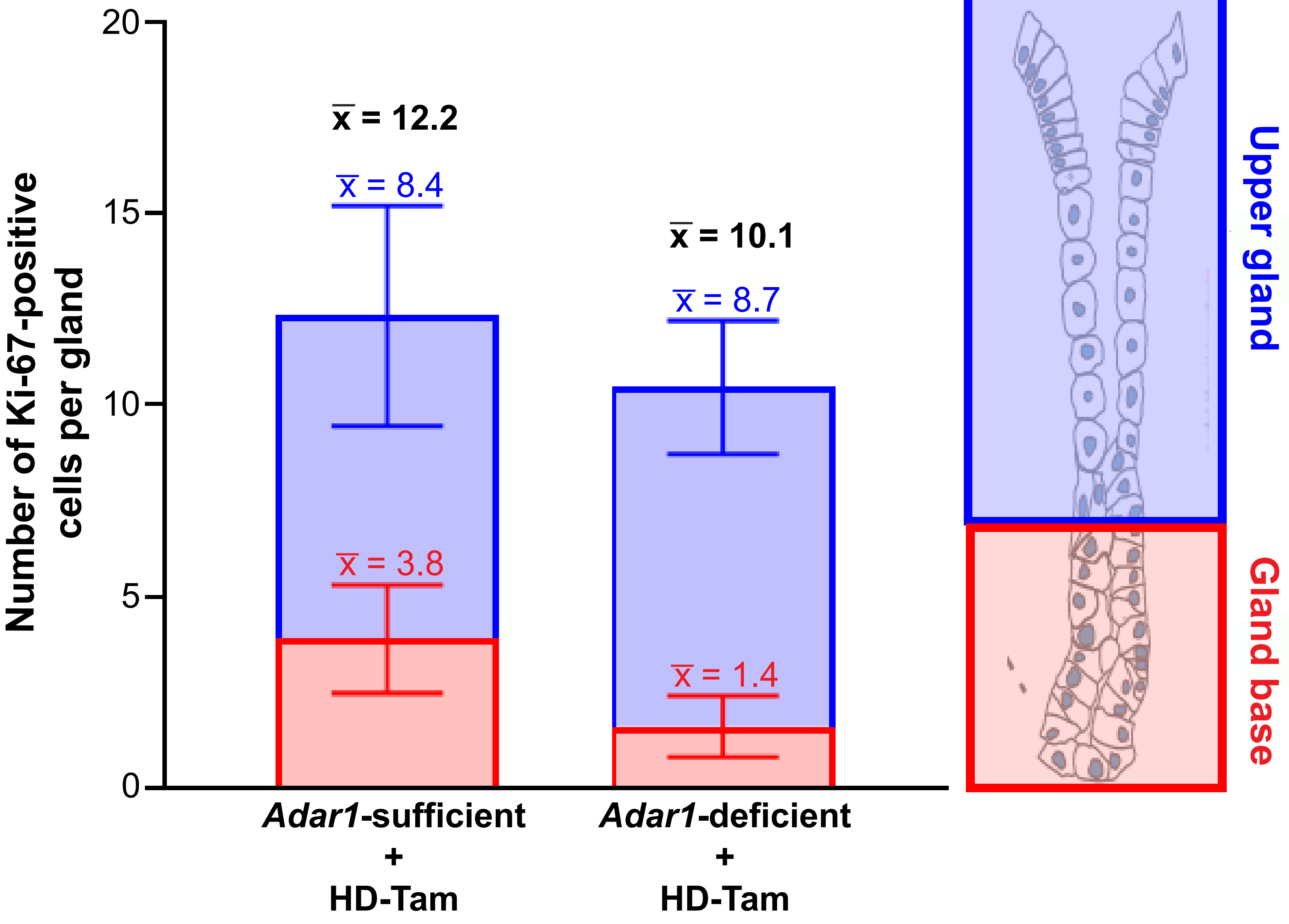


**Supplemental Figure 7. Chief cell-specific deletion of *Adar1* affects cellular proliferation at the gland base of metaplastic glands but has no effect on proliferation throughout the rest of the gland.** *Adar1$^{fl/fl}$;Mist1$^{Cre\text{-}ERT/+}$* mice were treated with vehicle or low-dose tamoxifen (LD-Tam) prior to inducing gastric metaplasia with high-dose tamoxifen (HD-Tam; see Figure 5A). The mean (±SEM) number of Ki-67-positive cells in pre-defined gland regions (see diagram on right) was determined from randomly selected fields across three biological replicates. The mean for each region is denoted by $\bar{x}$. The mean total number of Ki-67-positive cells across the entire gland is shown in black above each bar graph.

# Full unedited gels for Figures 2C and 2D

C

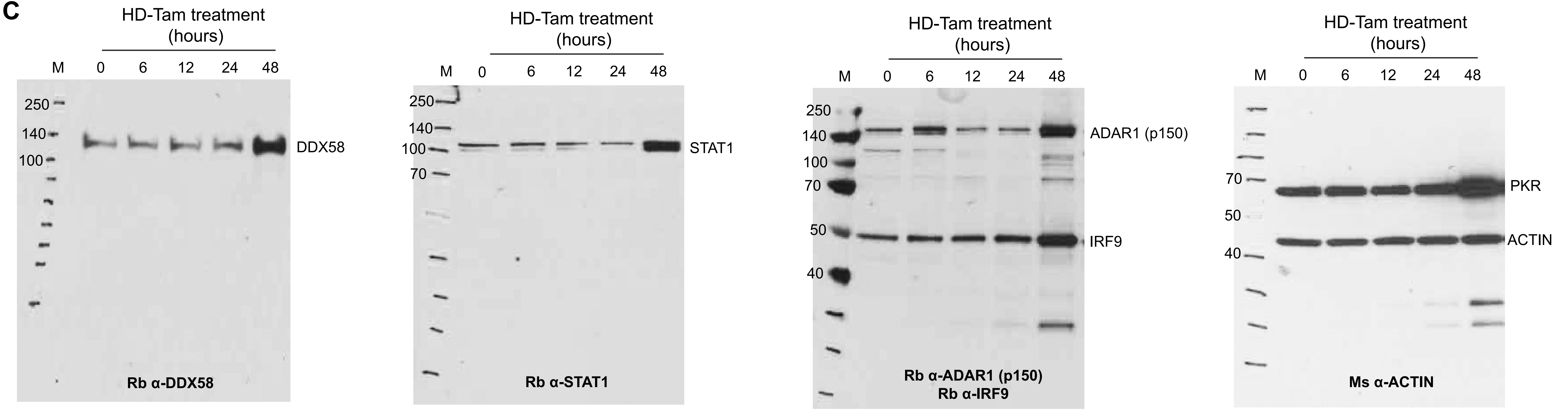


D

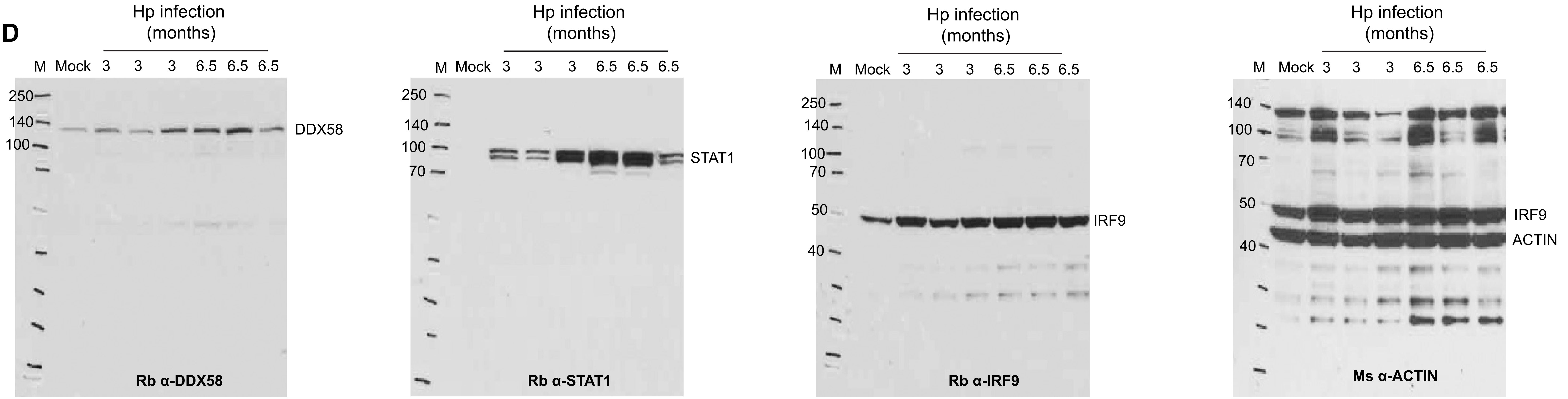

# Figure 3

**B**

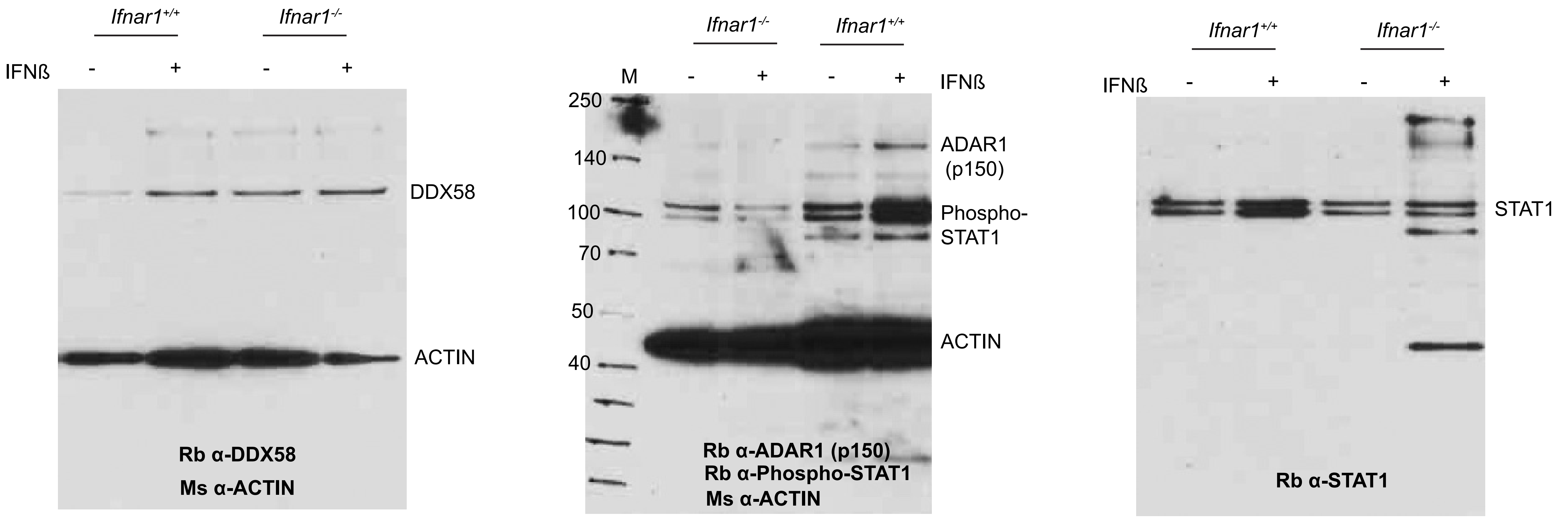
Ifnar1+/+
Ifnar1-/-
IFNß - + - +
DDX58
ACTIN
Rb α-DDX58
Ms α-ACTIN
Ifnar1-/-
Ifnar1+/+
M - + - + IFNß
250
140
100
70
50
40
ADAR1 (p150)
Phospho-STAT1
ACTIN
Rb α-ADAR1 (p150)
Rb α-Phospho-STAT1
Ms α-ACTIN
Ifnar1+/+
Ifnar1-/-
IFNß - + - +
STAT1
Rb α-STAT1

**G**

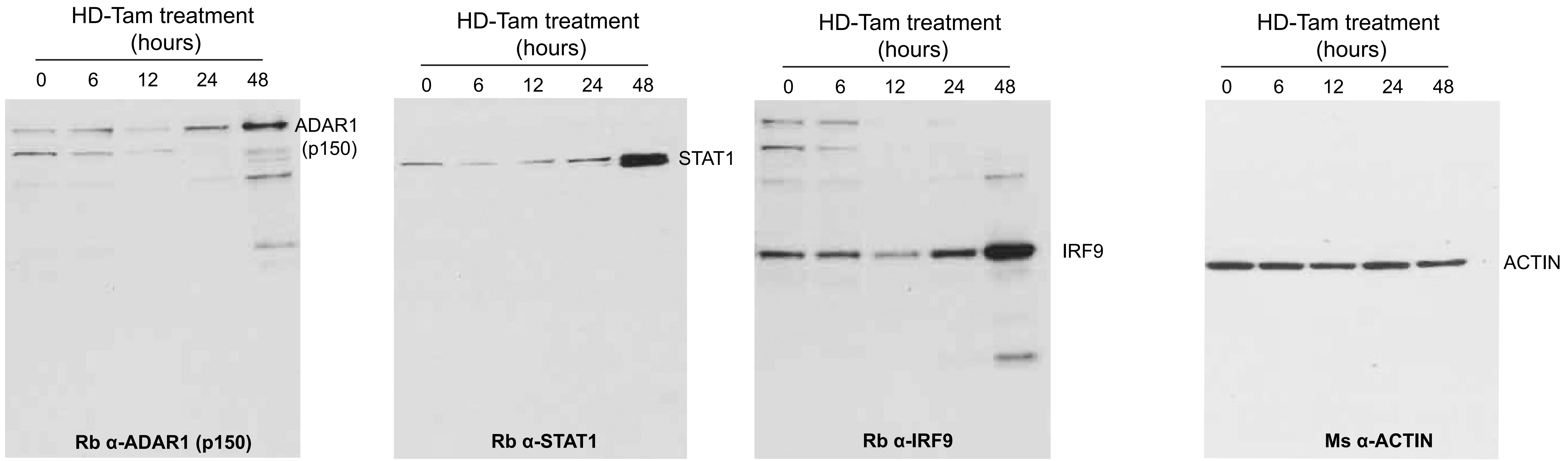
HD-Tam treatment (hours)
0 6 12 24 48
ADAR1 (p150)
Rb α-ADAR1 (p150)
HD-Tam treatment (hours)
0 6 12 24 48
STAT1
Rb α-STAT1
HD-Tam treatment (hours)
0 6 12 24 48
IRF9
Rb α-IRF9
HD-Tam treatment (hours)
0 6 12 24 48
ACTIN
Ms α-ACTIN

# Full unedited gels for Figures 4A, 4B, and 4E

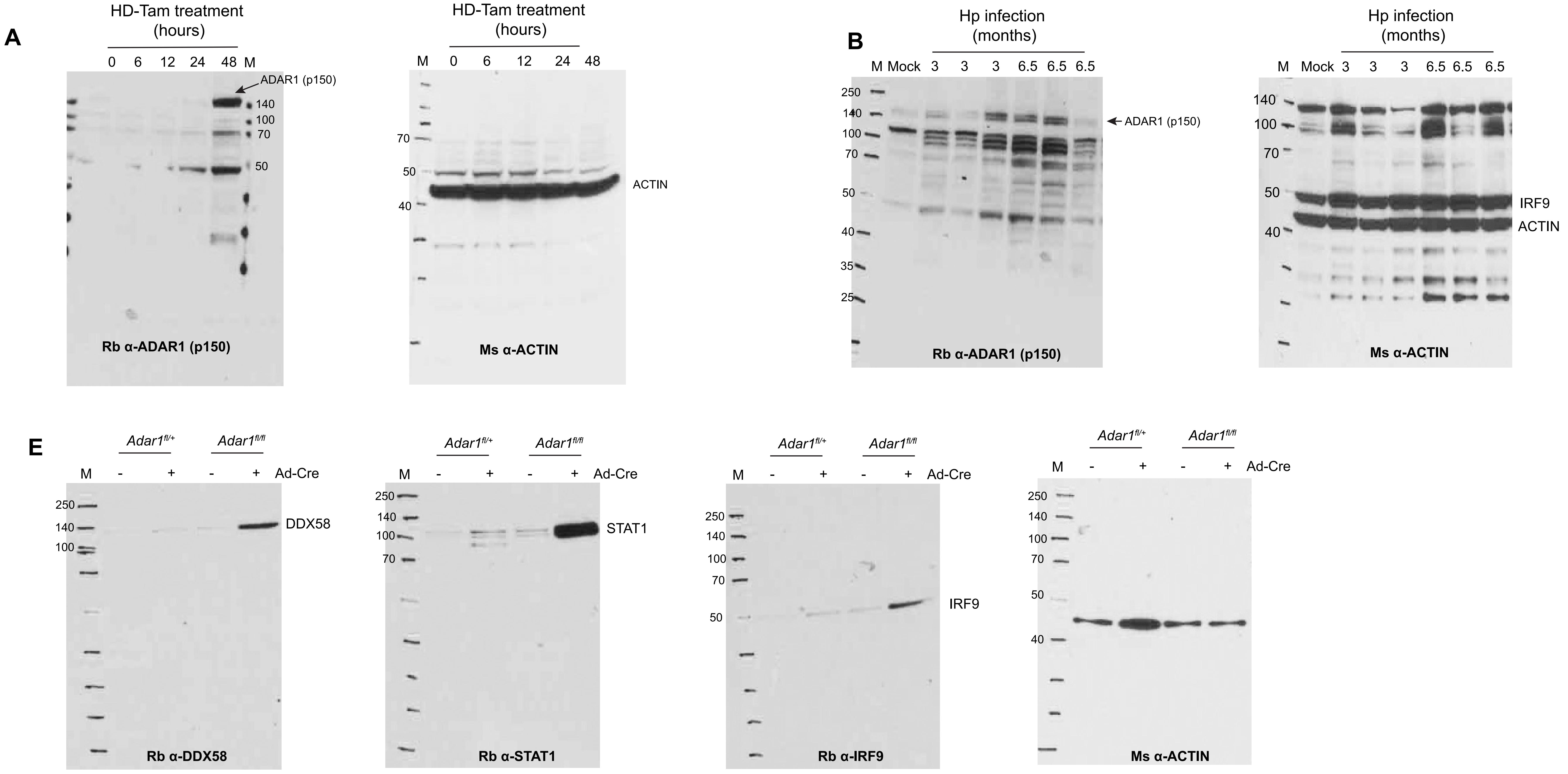

# Full unedited gels for Figure 5E

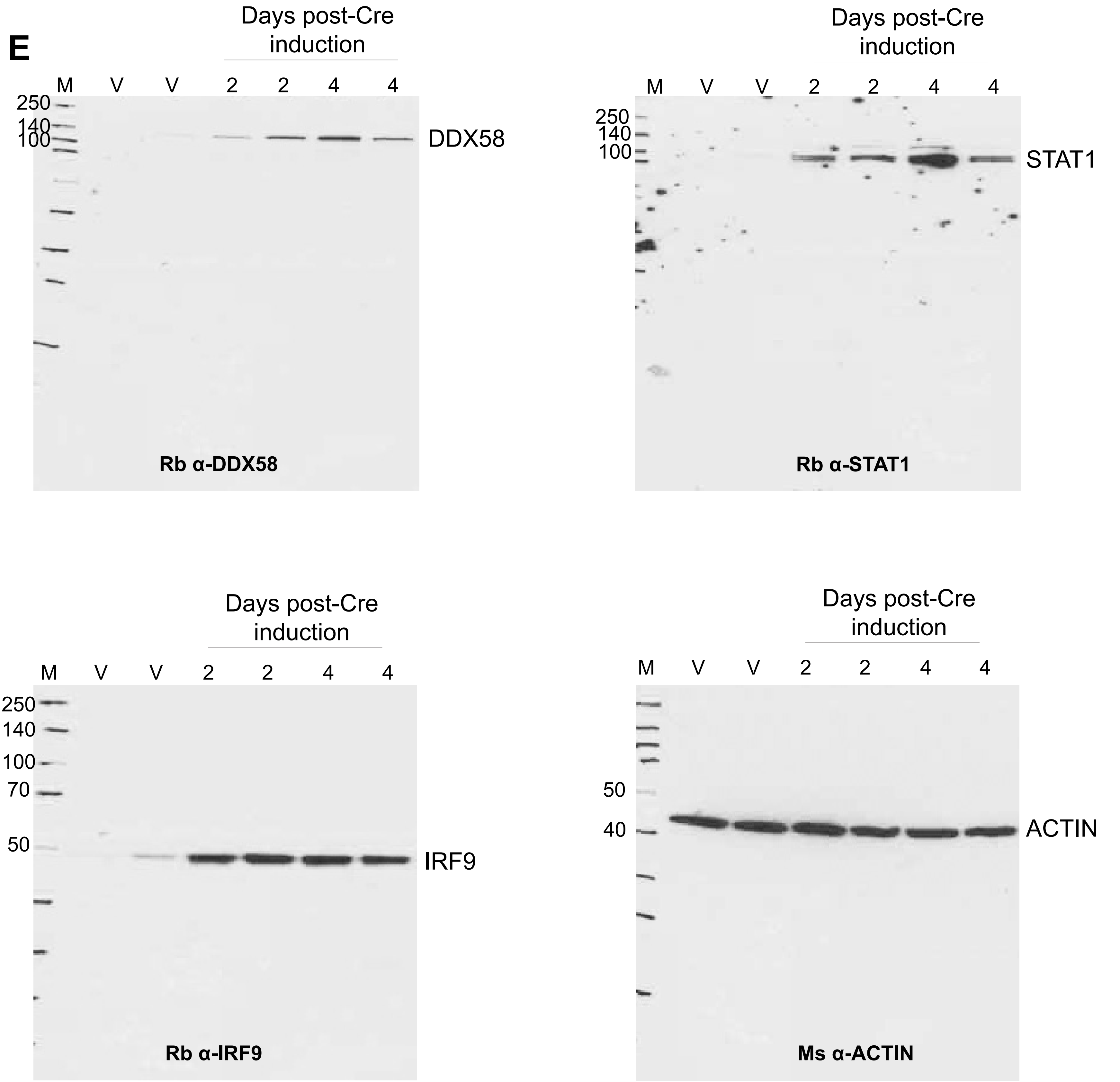

Supplement: Supplemental data [file jciinsight-7-153511-s082.pdf]
